# Encapsulation of Indicaxanthin-Rich *Opuntia* Green Extracts by Double Emulsions for Improved Stability and Bioaccessibility

**DOI:** 10.3390/foods13071003

**Published:** 2024-03-25

**Authors:** Sara Parralejo-Sanz, Isabel Quereda-Moraleda, Teresa Requena, M. Pilar Cano

**Affiliations:** 1 Laboratory of Phytochemistry and Plant Food Functionality, Biotechnology and Food Microbiology Department, Institute of Food Science Research (CIAL) (CSIC-UAM), Nicolás Cabrera 9, 28049 Madrid, Spainisa.que.mo@gmail.com (I.Q.-M.); 2 Laboratory of Functional Biology of Lactic Bacteria, Biotechnology and Food Microbiology Department, Institute of Food Science Research (CIAL) (CSIC-UAM), Nicolás Cabrera 9, 28049 Madrid, Spain; t.requena@csic.es

**Keywords:** *Opuntia ficus-indica* var. *Colorada*, indicaxanthin, betalains, phenolic compounds, encapsulation, double emulsions, physical stability, digestive stability, bioaccessibility

## Abstract

*Opuntia ficus-indica* var. *Colorada* fruit is an important source of indicaxanthin, a betalain with antioxidant, anti-inflammatory, and neuromodulatory potential, proven in both in vitro and in vivo models. Other betalains and phenolic compounds with bioactive activities have also been identified in *Colorada* fruit extracts. These compounds may degrade by their exposure to different environmental factors, so in the present research, two double emulsion systems (W_1_/O/W_2_) were elaborated using Tween 20 (TW) and sodium caseinate (SC) as surfactants to encapsulate *Colorada* fruit pulp extracts, with the aim of enhancing their stability during storage. Encapsulation efficiencies of up to 97.3 ± 2.7%, particle sizes between 236 ± 4 and 3373 ± 64 nm, and zeta potential values of up to ∣46.2∣ ± 0.3 mV were obtained. In addition, the evaluation of the in vitro gastro-intestinal stability and bioaccessibility of the main individual bioactives was carried out by standardized INFOGEST© protocol, obtaining the highest values for the encapsulated extract bioactives in comparison with the non-encapsulated extract (control). Especially, TW double emulsion showed bioaccessibility values of up to 82.8 ± 1.5% for the main bioactives (indicaxanthin, piscidic acid, and isorhamnetin glucoxyl-rhamnosyl-pentoside 2 (IG2)), indicating a promising potential for its use as a functional natural colorant ingredient.

## 1. Introduction

Cactus prickly pear fruits (*Opuntia* spp.) come from the arbustive plant called nopal, which belongs to the *Cactaceae* botanical family. To date, nearly 300 species of the genus *Opuntia* are known, with *Opuntia ficus-indica* being the most produced, traded, and consumed worldwide. This species is mainly grown in harsh and arid areas, due to its easy adaptation to dry environments and extreme climatic conditions [1].

Mexico is the main producer and exporter of *Opuntia ficus-indica*, but many other countries are usual consumers of prickly pears, i.e., Spain, China, South Africa, and Australia, among others [2]. In Spain, it is mainly grown in the Canary Islands, and even though it is a seasonal fruit that can only be harvested from June to September, it can be available until December. There are many different varieties of *Opuntia ficus-indica* cactus pears, which are usually classified based on their pulp color (white, yellow, red, orange, etc.). These color differences are mainly determined by the presence of phytochemical pigments, especially betalains [3].

Betalains are water-soluble, nitrogen-containing, vacuolar molecules that are synthesized as secondary plant metabolites that respond to adverse exogenous conditions [4]. They can be classified into two groups depending on their structural interaction with betalamic acid: betaxanthins, which are formed by conjugation with different amino acids and provide yellow-orange tones, with indicaxanthin (proline-betaxanthin) being the most abundant, as well as betacyanins, which are formed by condensation with cyclo-dopa or its glycoside derivatives and give rise to red-violet colorings, of which betanin stands out [5]. Betalains are recognized for their free radical scavenging and antioxidant activity and current research evidence has demonstrated their involvement in antimicrobial, anticancer, hepatoprotective, neuroprotective, and cardioprotective processes [6,7]. However, the chemical stability of betalains can be compromised by various environmental factors (pH, oxygen, elevated temperatures, light exposure, etc.) and, from a nutritional point of view, by the gastro-intestinal digestion process. This can lead to a decrease in their bioaccessibility and therefore in bioavailability, defaulting in their arrival to the target tissues in the organism and also diminishing their bioactivity. Different studies have reported that betalain degradation occurs mainly at the gastric and intestinal levels, reducing their absorption in the small intestine [8].

As previously mentioned, indicaxanthin is one of the most abundant betaxanthins, but its source is limited to *Opuntia ficus-indica* cactus prickly pears, *Rivina humili* (coral berry fruits), and *Myrtillocactus geometrizans* (garambullo berries), thereby being the reason why it has been less studied than betanin, as the latter is highly concentrated in *Beta vulgaris* (beetroot), which is broadly cultivated and consumed worldwide [9]. However, indicaxanthin has been proven to exert antioxidant, anti-inflammatory, antiproliferative, neuromodulatory, and anti-obesity-related dysmetabolic conditions effects in both in vitro and in vivo models [9], making it a very interesting and promising functional natural ingredient. In addition, it has been demonstrated to possess a quite large (>50%) non-polar surface that provides it with an amphipathic character. This could facilitate the intestinal absorption of this molecule regarding others betalains, due to its easy access into cells and its incorporation into the brain–blood barrier (study carried out in rat brains) [4]. Gómez-Maqueo et al. [3] analyzed indicaxanthin’s bioaccessibility from *Opuntia ficus-indica* fruit extracts and reported values of 58 ± 5% for *Colorada* (yellow-orangish variety) and of 70.7 ± 3.2% for *Morada* (purple variety), while Montiel-Sánchez et al. [10] reported values of 15 ± 9% for indicaxanthin from *Myrtillocactus geometrizans* berries. These differences can be associated with the different plant matrix and composition of each fruit species, which make it necessary to perform an individual analysis of each.

The pharmaceutical and food industries have keen interest in bioactive compounds from natural sources such as indicaxanthin, as they can improve the functional properties of the product (for example, in the case of indicaxanthin, as a natural colorant) and exert health benefits by their consumption, increasing marketing opportunities and consumer demand [11]. However, as previously mentioned, these types of compounds can degrade due to different factors, so various strategies have been developed in the past years to promote their protection, with the aim of extending their shelf life and improving their bioavailability. One of the most common techniques is encapsulation by water-in-oil-in-water double emulsions (W_1_/O/W_2_), which consist of complex disperse systems surrounding liquid droplets by a bilayer in which both hydrophilic and hydrophobic compounds can be co-encapsulated [12].

W_1_/O/W_2_ systems are composed of three phases: the primary aqueous phase (W_1_), in which the compounds of interest are dissolved, surrounded by the oil phase (O), which is responsible for maintaining the structure of the primary emulsion (W_1_/O), and, lastly, the external continuous phase, which in this case is the secondary water phase (W_2_). W_1_/O is dispersed into the W_2_, obtaining the complete W_1_/O/W_2_ system [13]. These systems are often elaborated with the aid of surfactants and emulsifiers, which are amphiphilic chemical agents that help reducing the interfacial tension between two immiscible phases, favoring droplet formation, and so providing the emulsion system with a higher stability [14]. W_1_/O/W_2_ emulsions are commonly used for the encapsulation of vitamins, minerals, and other desired compounds with hydrophilic properties. Some authors have reported different encapsulation methods for betalains from beetroot and purple *Opuntia ficus-indica* cactus fruits by double emulsion systems [15,16,17], but, to the best of our knowledge, there are no reported methodologies of encapsulation by double emulsions of orange or yellowish *Opuntia ficus-indica* cactus fruits abundant in indicaxanthin.

For the present study, extracts from *Opuntia ficus-indica* var. *Colorada* (OFC) pulps were encapsulated by two different water-in-oil-in-water (W_1_/O/W_2_) double emulsion systems. This fruit was selected as the matrix of study because of its high content in indicaxanthin, as previously reported by Gómez-Maqueo et al. [3], among other betalains and phenolic compounds, especially phenolic acids (from which piscidic acid is the most abundant) and flavonoids (in particular, isorhamnetin and quercetin glycosides) that have also demonstrated in multiple investigations an interesting biological potential [18]. The first double emulsion system (TW) was elaborated with polyglycerol poliricinoleate (PGPR) as the lypophilic emulsifier and Tween 20 as the hydrophilic emulsifier, which are well known for their stabilizing properties, and glycerol as the co-adjuvant [14,19]. For the second double emulsion system (SC), PGPR was also used as the lypophilic emulsifier, and sodium caseinate as the hydrophilic emulsifier, as it has been demonstrated that some proteins, due to their amphiphilic nature, can serve as natural emulsifying agents by lowering the interfacial tension between oil and water phases [20].

A detailed individual examination was conducted to assess the encapsulation efficiency of indicaxanthin, along with the other individual and total betalains and phenolic compounds co-extracted from *Opuntia ficus-indica* var. *Colorada* fruit pulps, using the proposed double emulsion systems. Additionally, the physical characteristics of these formulations were measured to determine their stability during storage, including factors such as creaming, particle size, and electrostatic interactions. Furthermore, a comprehensive evaluation of the gastro-intestinal stability and bioaccessibility of the individual compounds was undertaken through the in vitro digestion INFOGEST© standardized protocol. This investigation presents a potential contribution to the development of new, healthful ingredients abundant in natural compounds like indicaxanthin, featuring enhanced physico-chemical properties for the food and pharmaceutical industries.

## 2. Materials and Methods

### 2.1. Reagents, Solvents, and Standards

Ultra-pure MilliQ water was obtained from a Millipak^®^ Express 40 system MerkMillipore (Darmstadt, Germany). Ethanol (99.97%) was obtained from VWR International (Barcelona, Spain). Formic acid was purchased from Panreac Química (Barcelona, Spain). Sephadex LH-20, standards (isorhamnetin, quercetin, rutin, 4-hydroxybenzoic acid) and amino acids (glycine, asparagine, glutamine, glutamic acid, proline, and tryptophan) were purchased from Sigma-Aldrich (St. Louis, MO, USA). Betanin was extracted, isolated, and purified from lyophilized beetroots (*Beta vulgaris* subsp. *vulgaris*); betaxanthins were semi-synthesized from it. Piscidic acid was purified from prickly pear peels by semi-preparative high-performance liquid chromatography (HPLC) following the methodology reported by García-Cayuela et al. [21]. The standards of isorhamnetin glycosides were supplied from Dr. Serna-Saldivar from the Biotechnology Center FEMSA (Escuela de Ingeniería y Ciencias, Instituto Tecnológico de Monterrey, Monterrey, Mexico).

For the elaboration of the double emulsion systems (W_1_/O/W_2_), NaCl was purchased from Panreac Química S.L.U. (Barcelona, Spain). Tween 20 (polysorbate 20), glycerol, and Sodium Caseinate Salt were obtained from Sigma-Aldrich (St. Louis, MO, USA). Medium-Chain Triglycerides from Coconut Oil (MCT oil) were supplied by Ketosource Ltd. (London, UK). Buffer TRIS pH 9 (Tris (hydroxymethyl)aminomethane) was obtained from Merck KGaA (Darmstadt, Germany). Gelatin and arabic gum were purchased from Acros Organics (Geel, Belgium) and Guar Gum was obtained from MCS (Puebla, Mexico), while polyglycerol polyricinoleate (PGPR) was from Palsgaard A/S (Juelsminde, Denmark). Phosphatidylcholine (PC) was purchased from Avanti Polar Lipids (Alabaster, AL, USA). Lipase enzyme (RGE15) was purchased from Lipolytech (Marseille, France). Amylase (10080), Pepsin (P6887), Pancreatin (P7545), bile salts (B838), and other reagents used for the in vitro digestion assay were obtained from Sigma-Aldrich (St. Louis, MO, USA).

### 2.2. Plant Material and Physicochemical Analysis

**Figure 1:**
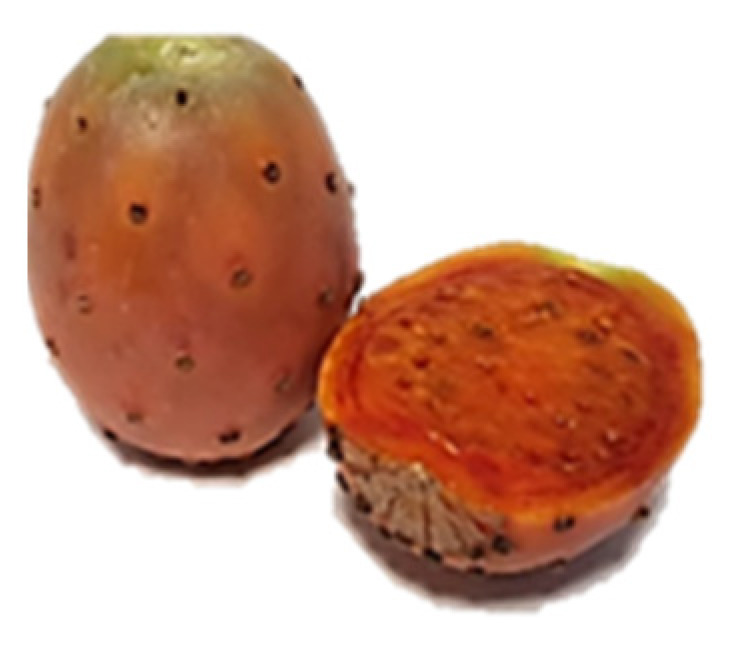
Image of *Opuntia ficus-indica* var. *Colorada* whole fruits from the Canary Islands (Spain).

*Opuntia ficus-indica* var. *Colorada* (OFC) fruits were harvested in 2022 from Fasnia (Tenerife, Canary Islands, Spain; 28°2′ N, 16°4′ W; 446 m above sea level) (Figure 1). Fruits were chosen attending to their color, size, maturity, and integrity, discarding the damaged ones. Physicochemical characteristics of the prickly pear fruits of *Opuntia ficus-indica* var. *Colorada* (OFC) were analyzed (Table 1). Titratable acidity (g of citric acid/100 g of fresh pulp) was measured by neutralization of the pulps’ juice with 0.1 N sodium hydroxide until an 8.1 pH was reached. pH and soluble solids (°Brix) were determined from the pulps’ juice too. Color of the pulps was characterized by the CIELAB scale (L*: lightness; a*: green-red tonality; b*: blue-yellow tonality) using a Konica Minolta CM-3500d (Tokyo, Japan). After this, *Colorada* fruits were peeled under diminished light conditions, and the pulps were frozen with liquid nitrogen and lyophilized. After that, seeds were removed and the pulps were pulverized (Grindomix GM200, Retsch, Germany) to a fine particle size (<2 mm), vacuum-packed, and stored in the dark at −24 °C until use.

### 2.3. Obtention of Green Extracts Rich in Bioactive Compounds

Betalain- and phenolic-rich green extracts from the lyophilized pulps of *Opuntia ficus-indica* var. *Colorada* were obtained according to the methodology described by Gómez-Maqueo et al. [3] with modifications. One gram of freeze-dried, pulverized *Colorada* pulp was processed with 5 mL ethanol–water (1:1, *v*:*v*), homogenized with a vortex for 1 min, and treated for 4 min with an ultrasonic water bath (3,000,514, 50/60 Hz, 360 W, J.P Selecta S.A., Barcelona, Spain) for each sample. Then, samples were centrifuged for 10 min at 10,000× *g* at 4 °C, supernatants were collected, and the extraction step was performed two more times with 3 mL ethanol–water (1:1, *v*:*v*) and one last time with pure ethanol. All the supernatants were gathered in rounded flasks to evaporate the ethanolic fraction in a rotary evaporator (Buchi, Flawil, Switzerland) at 30 °C. Finally, the obtained residues (extracts) were lyophilized (LyoBeta 15Azbil Telstar SL, Terrasa, Spain), rinsed up to 5 mL with ultrapure MilliQ water, filtered with 0.22 µm syringe nylon filters (E0032, Análisis Vínicos, Spain), and analyzed by high-performance liquid chromatography (HPLC). Extract samples were also lyophilized (LyoBeta 15Azbil Telstar SL, Terrasa, Spain) to use them for the encapsulation assays.

### 2.4. Analysis of Betalains and Phenolic Compounds by HPLC-DAD-MS

Total and individual betalains and phenolic compounds from *Opuntia ficus-indica* var. *Colorada* extracts were characterized and quantified according to the method reported by Gómez-Maqueo et al. [3]. The 1200 Series Agilent HPLC-DAD System (Agilent Technologies, Barcelona, Spain) equipment with a C18, reverse column Zorbax SB-C18, 250 × 4.6 nm i.d., S-5 µm (Agilent Technologies, Santa Clara, CA, USA) at 25 °C was used. Phase A was composed of ultrapure water with 1% formic acid (*v*/*v*) and Phase B was composed of methanol (99.8% LC-MS) with 1% formic acid (*v*/*v*). Both were used in gradient for 70 min to obtain an optimal separation of the bioactive compounds. The volume of injection used was 20 µL and a flow rate of 0.8 mL/min was utilized. The photodiode array detector that was UV-Visible was set at four wavelengths: 535 nm for betacyanins, 480 nm for betaxanthins, 370 nm for flavonoids, and 280 nm for phenolic acids. The HPLC-DAD was coupled to a mass spectrometry detector (LCMS SQ 6120, Agilent, Agilent Technologies, Santa Clara, CA, USA) with an electrospray ionization (ESI) source operating in positive ion mode. The drying gas was nitrogen at 3 L/min at 137.9 KPa. The nebulizer temperature was 300 °C and the capillary had 3500 V potential. The coliseum gas was helium, and the fragmentation amplitude was 70 V. Spectra were recorded *m*/*z* from 100 to 1000. In addition, mass spectrometry analysis was performed in the LC-QTOF equipment (Bruker Daltonics, Bremen, Germany) with an ESI source and the same chromatographic conditions previously mentioned. The ESI-QTOF detector worked in positive ion mode and recorded mass spectra from 50 to 3000. The analysis conditions applied were 300 °C, a capillary voltage of 3500 V, a charging voltage of 2000 V, a nebulizer at 2.0 bar, and dry gas in a flow rate of 6 L/min. The bbCID method (Broad Band Collision Induces Dissociation) was used for the MS/MS analysis.

Each compound was identified according to their retention times, UV/Vis, and mass spectra in comparison to the purified, semi-synthesized, or commercial standards. Betaxanthins (indicaxanthin, portulacaxanthins, and vulgaxanthins), betanin, piscidic acid, and isorhamnetin glycosides (IG1 and IG2) were determined using the calibration curves of their individual standards. Quercetin glycosides were quantified using the calibration curve of quercetin and 4-hydroxybenzoic acid derivative was quantified using the calibration curve of hydroxybenzoic acid.

### 2.5. In Vitro Antioxidant Capacity of Opuntia ficus-indica var. Colorada Pulp Green Extracts

For the in vitro determination of the antioxidant activity of the obtained OFC pulp green extracts, the ORAC (oxygen radical absorbance capacity) method was carried out according to the methodology of Ou et al. [22], which measures the loss of fluorescence of the samples by the action of 2,2′-azobis (2-amidinopropane) dihydrochloride (AAPH) that degrades the fluorescent compound. Each well assayed contained 75 mM Na-phosphate buffer (pH 7.4), 7 µM fluorescein, and an appropriate volume of sample or blank, reaching a final volume of 0.2 mL. The reaction was started by adding 46 mM AAPH on each well and the loss of fluorescence was measured every minute during 90 min at 37 °C using two wavelengths (485 nm for excitation and 530 nm for emission). To quantify the antioxidant capacity, the difference between the area under the fluorescence decay kinetic curve (area under curve, AUC) of the sample and the AUC of the blank was calculated. Antioxidant capacity was determined using a Trolox dose–response curve (10–80 µM).

### 2.6. Elaboration of Double Emulsion Systems W_1_/O/W_2_

Lyophilized OFC extracts were encapsulated using two double emulsion systems (W_1_/O/W_2_) that were named TW, referring to the system based on Tween 20 and SC, which contained sodium caseinate (Table 2). Both were elaborated following the methodology described by Parralejo-Sanz et al. [23] with modifications. The composition in reagents and ingredients used to prepare TW and SC double emulsions can be seen in Table 2.

#### 2.6.1. Tween 20-Based W_1_/O/W_2_ Double Emulsion Systems (TW)

To obtain the double emulsion systems based on Tween 20 (TW) the primary emulsion (W_1_/O) was formed first. The aqueous phase (W_1_) constituted 0.1 M NaCl solution, in which OFC extract was dissolved and glycerol as co-surfactant. Three different OFC extract quantities were used: emulsion TW1, which contained 1 g of OFC pulp extract; TW2, with 2 g of OFC pulp extract; and TW3, with 3 g of OFC pulp extract (Table 2). The oil phase (O) was composed of MCT oil and polyglycerol polyricinoleate (PGPR). W_1_ phase was dispersed drop-wise into the oil phase using an Ultra-Turrax T-25 digital homogenizer (IKA Works Inc., Staufen im Breisgau, Germany) at 7200 rpm for 10 min (4:2:4 min, ON:OFF:ON) with an external ice-water bath to avoid overheating, and they continuously were treated with an ultrasound sonifier (Sonifier digital SFX 550, Branson, MO, USA) using a 23 mm probe (Biogen Científica S. L., Madrid, Spain) and a 3 min cycle at 70% amplitude with 1 s ON: 1 s OFF pulses, as well as with external cooling by ice-water bath obtaining the complete primary emulsion (W_1_/O).

Then, double emulsion was prepared including W_1_/O on the secondary water phase (W_2_). W_2_ was composed of a 0.1 M NaCl solution and the surfactant Tween 20. The dispersion of W_1_/O into W_2_ was achieved using the Ultra-Turrax T-25 homogenizer (IKA Works Inc., Staufen im Breisgau, Germany) at 3600 rpm for 5 min (2:1:2, min, ON:OFF:ON); afterwards, the Sonifier with a 23 mm diameter ultrasound probe at a 60% amplitude during 90 s in continuous pulse mode with an external ice-water bath. The obtained double emulsions were stored at 7 °C for the physical stability studies.

#### 2.6.2. Sodium Caseinate-Based W_1_/O/W_2_ Double Emulsion Systems (SC)

The double emulsion systems based on sodium caseinate (SC) were also obtained preparing firstly the simple inner emulsion. W_1_ was prepared dissolving OFC extract in a solution of 6% gelatine in TRIS buffer (pH 9). Again, three different quantities of OFC extracts were used: 1 g for SC1, 2 g for SC2, and 3 g for SC3 (Table 2). The oil phase constituted MCT oil, PGPR, and phosphatidylcholine (PC). First, the oil phase (O) was homogenized for 1 min using an Ultra-Turrax T-25 homogenizer at 4000 rpm and, immediately, W_1_ was dispersed into it by using the Ultra-Turrax T-25 homogenizer again at 16,000 rpm, for 3 cycles of 1 min:1 min (ON: OFF) with an external ice-water bath. Then, samples were treated with the ultrasound equipment with a 23 mm diameter ultrasound probe, running at a 30% amplitude for 2 cycles of 30 s (1 s:1 s, ON: OFF), with external ice-water bath.

Continuously, the double emulsion (W_1_/O/W_2_) was obtained dispersing the primary emulsion into the secondary water phase, which was composed of a 3% sodium caseinate solution, glycerol with 10% of NaCl 0.1 M, arabic gum, and guar gum. First, the W_2_ phase was homogenized for 1 min using the Ultra-Turrax T-25 homogenizer at 4000 rpm. Then, it was mixed with the primary emulsion (W_1_/O) using again the Ultra-Turrax T-25 homogenizer at 11,000 rpm during 2 min in continuous pulse mode. The obtained double emulsions were stored at 7 °C for the physical stability studies.

### 2.7. In Vitro Gastro-Intestinal Digestion Assay

The in vitro gastro-intestinal digestion assay was performed following the INFOGEST^®^ standardized protocol [24], where OFC pulp non-encapsulated extracts were used as control and the freshly prepared double emulsion systems on the different defined concentrations of extract as test samples. The composition of the in vitro gastro-intestinal digestion phases (oral, gastric, and intestinal) is shown in Table S1 (Supplementary Materials). The addition of the digestive enzymes into the different fluids was carried out just before the beginning of the assay. To stop the possible occurring reactions, immediately after the end of each phase, samples were filtered with 0.22 µm syringe filters (E0033, Análisis Vínicos, Ciudad Real, Spain) into amber vials and were frozen and stored at −20 °C. Betalains and phenolic compounds (phenolic acids and flavonoids) from each digestion phase were analyzed as previously described in Section 2.4. Digestive stability (µg of compound/sample) and recovery percentage of each individual compound was calculated comparing the initial concentration in the non-digested samples and at the end of the different digestion phases. Bioaccessibility was calculated using the following equation:

$$\mathrm{Bioaccessibility}\;\left(\%\right)=\frac{\mathrm{Concentration}\;\mathrm{of}\;\mathrm{compound}\;\mathrm{in}\;\mathrm{the}\;\mathrm{intestinal}\;\mathrm{phase}}{\mathrm{Initial}\;\mathrm{concentration}\;\mathrm{of}\;\mathrm{compound}}\times 100$$

### 2.8. Characterization of Double Emulsions (W_1_/O/W_2_)

Double emulsion systems based on Tween 20 (TW) and sodium caseinate (SC) were obtained as explained on Section 2.4. After elaboration, samples were stored at 7 °C for 20 days and aliquots of each sample were taken in triplicate in 5 days intervals to characterize them.

#### 2.8.1. Encapsulation Efficiency

The encapsulation efficiency (%) of each individual bioactive compound was performed based on the method reported by Parralejo-Sanz et al. [23]. Double emulsion systems (TW and SC) were centrifuged (Centrifuge 5804R, Eppendorf, Hamburg, Germany) at 12,880× *g* for 15 min at 4 °C for TW double emulsions and at 2608× *g* during 10 min at 4 °C for SC double emulsions. Then, the supernatants were weighted and mixed with 5 mL of chloroform–methanol–water mixture 60:10:30 (%, *v*/*v*/*v*) for TW, and the same solvents but in 67:23:10 proportion (%, *v*/*v*/*v*) were used for SC. Continuously, these mixtures were centrifuged for 3 min under the same conditions and the aqueous phases were removed. Samples were then placed on a rotary evaporator (Buchi, Flawil, Switzerland) at 30 °C to remove the solvents, filtered with 0.22 µm syringe filters (E0033, Análisis Vínicos, Ciudad Real, Spain) into amber vials, and stored at −20 °C until their analysis. The concentration of each encapsulated compound was compared with the concentration of the non-encapsulated extract, thereby obtaining the encapsulation efficiency. The following equation was used:

$$\mathrm{Encapsulation}\;\mathrm{Efficiency}\;\left(\%\right)=\frac{\mathrm{Concentration}\;\mathrm{of}\;\mathrm{bioactive}\;\mathrm{encapsulated}\;}{\mathrm{Concentration}\;\mathrm{of}\;\mathrm{bioactive}\;\mathrm{on}\;\mathrm{initial}\;\mathrm{extract}}\times 100$$

#### 2.8.2. Optical and Confocal Microscopy Study of TW and SC Double Emulsions

Optical microscopy images of TW and SC double emulsions were obtained to confirm the correct formation and droplet structure of the different systems. A vertical microscope Axioskop (Carl Zeiss, Oberkochen, Germany) with a Zeiss Plan-Neofluar lens (Carl Zeiss, Göttingen, Germany) coupled to a Leica DMC 6200 pixel-shift camera (Leica Microsystems, Wetzlar, Germany) was used. Samples were prepared by depositing one drop of each emulsion on microscope slides, without using stains. Samples were observed at 40× and 100× using an open condenser and illumination (level four).

Confocal laser scanning microscopy analysis was also performed to evaluate the microstructure of the emulsion particles and their progression during the in vitro gastro-intestinal digestion assay. A droplet of each sample was placed on a microscope slide and stained with 10 µL of Nile red solution (1 mg of Nile red/mL of acetone). Fluorescence excitation and emission were measured at 488 and 523–650 nm, respectively. Then, samples were studied with a confocal multispectral TCS SP5 system (Leica Microsystems, Germany) at 20× and at 40× using a Zeiss Plan-Neofluar lens.

#### 2.8.3. Physical Stability of Double Emulsions during Cold Storage

The analysis of physical stability of TW and SC double emulsions (W_1_/O/W_2_) was performed according to Hong et al. [25]. The stability was expressed in mm of creaming (gravitational phase separation), and it was measured in triplicate during sample storage at 7 °C for 20 days.

#### 2.8.4. Particle Size and Zeta Potential

Particle size, particle size distribution, polydispersity index, and zeta potential of TW and SC double emulsions were analyzed using a Zetasizer Pro Equipment (Malvern Instruments Ltd., Worchestersire, UK).

For these analyses 100 µL of each sample were diluted on 10 mL of ultrapure MilliQ water, homogenized at 400 rpm using a stirring plate, and let to stand for 5 min prior to analysis. Then, samples were placed on the equipment using folded capillary cells, which were left equilibrating for 2 min at 25 °C. The analyses were carried out using a scattering angle of 174° and a refraction index of 1.47. Data were expressed in nm and mV for particle size and zeta potential, respectively.

### 2.9. Statistical Analysis

Data were expressed as mean ± standard deviation of at least three independent determinations (*n* = 3). Significant differences at a *p* < 0.05 significance level were calculated by an analysis of variance (ANOVA) with the SPSS Statistics software 26.0 for Windows (IBM corp., Armonk, New York, NY, USA). In addition, a Tukey test was used to make all of the pairwise comparisons between groups or samples.

## 3. Results and Discussion

### 3.1. Composition in Main Bioactives of O. ficus-indica var. Colorada Pulps Extracts and TW and SC Double Emulsions

The characterization data of the main identified total and individual betalains and phenolic compounds of prickly pear fruit *Colorada* pulps green extracts is shown in Table 3, while the quantification data of each compound in OFC extract and also in the double emulsion systems (TW and SC) with encapsulated OFC extract are shown in Table 4.

In addition, the HPLC-DAD chromatograms that were detected at 535 nm (for betacyanins), at 480 nm (for betaxanthins), at 370 nm (for flavonoids), and at 280 nm (for phenolic acids) for the OFC pulp green extract and for the encapsulated extract by TW2 double emulsion are shown in Figure S1 (Supplementary Materials).

A total of six betalains (five betaxanthins and one betacyanin (betanin)) and seven phenolic compounds (two phenolic acids and five flavonoids) were identified in the OFC pulps green extracts, while in the double emulsion systems (TW and SC) with encapsulated OFC extract, only betalains from the family of betaxanthins were detected (five betaxanthin compounds) as well as only five phenolic compounds (two phenolic acids and three flavonoids), because betanin, quercetin glycoside 1 (QG1), and isorhamnetin glucoxyl-rhamnosyl-rhamnoside (IG1) were not detected, as shown in Figure S1 (Supplementary Materials).

Indicaxanthin was the most abundant betalain in OFC extracts (508 ± 10 µg/g d.w.) as well as in TW double emulsions (from 243 ± 34 to 1366 ± 16 µg/emulsion) and SC double emulsions with OFC extract encapsulated (from 257 ± 20 to 1444 ± 170 µg/emulsion), depending on the amount of OFC-encapsulated extract in them (Table 4).

The observed indicaxanthin content in OFC pulp in the present study was similar to that of the data reported by Gómez-Maqueo et al. [3], who also analyzed Colorada fruits and showed that indicaxanthin was the most abundant betalain (11.79 ± 0.15 mg/100 g f.w.) of fresh fruit. Tesoriere et al. [26] also reported an indicaxanthin content of 12.32 ± 1.01 mg/100 g f.w. in the Sicilian yellow variety of *Opuntia ficus-indica* fruits, which is similar to the amount found in the Spanish *Colorada* fruit variety. In addition, comparing *Colorada* with other Spanish *Opuntia ficus-indica* prickly pear varieties (red, white, and purple), *Colorada* was the one with the highest content in indicaxanthin [27].

With respect to the content in phenolic compounds, piscidic acid was the most abundant one in OFC pulp extract (2641 ± 92 µg/g d.w.), in TW double emulsion with OFC extract (from 1694 ± 75 to 11,449 ± 156 µg/emulsion), and in SC double emulsion with OFC extract (from 1614 ± 176 to 11,345 ± 436 µg/emulsion). Gómez-Maqueo et al. [6] quantified the individual phenolic compounds present in *Colorada* pulps and piscidic acid was also the most abundant phenolic compound (4749 ± 5 µg/g d.w.). The differences in the bioactive amount among the data obtained in the present work and those previously published on the OFC fruits could be due to the fact that the composition of the fruits could vary seasonally, depending on the harvest year, the agronomic conditions, and climate, as well as because of small differences in the maturity of fruits in the moment of harvesting.

### 3.2. Antioxidant Capacity of O. ficus-indica var. Colorada Pulps Extracts

Once the individual and total content of betalains and phenolic compounds present in *Opuntia ficus-indica* var. *Colorada* pulps green extracts was evaluated, the antioxidant capacity was measured by the ORAC (Oxygen Radical Antioxidant Capacity) methodology to assess the biological potential of this extract related to its composition and to know the remaining antioxidant capacity of encapsulated extracts.

In the present study, the antioxidant capacity of OFC extract was 58.5 ± 4.1 µmol Trolox eq/g d.w. Recently, our research group published a study focused on the antioxidant potential of different *Opuntia* spp. varieties (including *O. ficus-indica* var. *Colorada*) and of some of the main individual isolated bioactives present in this extract, analyzing the different in vitro methodologies of LOX-FL (lipoxygenase-fluorescein), ORAC, and TEAC (Trolox Equivalent Antioxidant Capacity) (Table S2, Supplementary Materials) [6]. In this published study, different sensitivities were obtained for the different families of bioactive compounds, depending on the method employed. These authors reported antioxidant capacity values for *Opuntia ficus-indica* var. *Colorada* pulp extracts of 51.6 ± 1.9 µmol Trolox eq./g d.w. for the ORAC method and of 48.4 ± 2.1 µmol Trolox eq./g d.w. using the TEAC method, so even though these analyses were carried out at different times, the results were similar to those obtained in the present work. In contrast, OFC extracts showed values of 3.7 ± 0.2 µmol Trolox eq./g d.w. by the LOX-FL methodology, but when the antioxidant capacity of isolated indicaxanthin was analyzed by this method, values of up to 104 ± 2 µmol Trolox eq./g d.w. were obtained. In contrast, the results obtained for indicaxanthin with the ORAC and TEAC methods were significantly lower (17.3 ± 0.7 and 9 ± 2.1 µmol Trolox eq./g d.w., respectively). These differences were related to the fact that the LOX-FL assay showed a better specificity to assess the antioxidant potential of betalains (indicaxanthin in this case). The inhibition curves of LOX-FL for the individual OFC extracts standards can be seen in Figure S2 (Supplementary Material). These results can be compared with those published by Kuti [28] who analyzed the antioxidant capacity of different *Opuntia* species by the ORAC methodology and reported values between 15.8 ± 1.6 and 49.2 ± 1.7 µmol Trolox eq./g d.w. In this aspect, it could be said that the bioactive compounds present in the extracts of the pulp of *Opuntia ficus-indica* var. *Colorada* present an interesting in vitro antioxidant potential, so their encapsulation may be beneficial to enhance their stability.

### 3.3. Characterization of the Double Emulsion Systems (W_1_/O/W_2_)

As mentioned above, double emulsion systems with encapsulated *Opuntia ficus-indica* var. *Colorada* extracts were stored for 20 days at 7 °C and samples were collected each five days to analyze their stability during storage. Encapsulation efficiency and in vitro gastro-intestinal digestion analysis were only performed with freshly prepared double emulsions with encapsulated OFC extract (day 0).

#### 3.3.1. Encapsulation Efficiency of the Main Bioactives Encapsulated by Double Emulsions (W_1_/O/W_2_)

Encapsulation efficiency (EE, %) refers to the quantity of a compound that is integrated in an encapsulation system in comparison with the total quantity of the compound. Values of individual encapsulation efficiency of the main betalain and phenolic compounds present in the encapsulated extracts obtained for the Tween 20 (TW)- and sodium caseinate (SC)-based double emulsion systems are shown in Table 5. The chromatograms obtained from the HPLC analysis of the encapsulated extracts by double emulsion system TW2 (in which 2 g of OFC pulp green extract is encapsulated) detected at 535 nm (for betacyanins), at 480 nm (for betaxanthins), at 370 nm (for flavonoids), and at 280 nm (for phenolic acids) are shown in Figure S1 (Supplementary Materials).

With regard to the families of the different bioactive compounds encapsulated (betalains, phenolic acids, and flavonoids), betalains were the most efficiently encapsulated compounds by the assayed double emulsions (Table 5). The double emulsion systems elaborated with Tween 20 showed the higher EEs% for total betalains, especially for TW2 (double emulsion system based on Tween 20, containing 2 g of OFC pulp green extract) with values of 84.4 ± 4.5%.

With respect to the encapsulation efficiency of the individual betalains, the EE% of indicaxanthin showed the highest values, in a range from 77.7 ± 5.1 to 97.2 ± 0.4, where TW2 (double emulsion system based on Tween 20, containing 2 g of OFC pulp green extract) was the best option in order to encapsulate this betalain. These results were similar to those reported by Carmona et al. [29], who used maltodextrin and cactus mucilages to encapsulate *Opuntia ficus-indica* yellow-orange prickly pear pulp extracts, reporting that the indicaxanthin Ees% ranged from 87 ± 0.4 to 100 ± 0.

Among total phenolic acids, TW2 double emulsion was again the most efficient system to encapsulate them, with the average Ees% of 75.4 ± 2.4. With respect to the individual phenolic acids and piscidic acid showed the highest encapsulation efficiencies (between 71.8 ± 6.4 and 97.3 ± 2.7), standing out again in TW2 double emulsion.

With respect to the encapsulation of OFC total flavonoid compounds by double emulsions, TW2 was also the most efficient system, showing Ees% of 71.4 ± 3.3. Regarding the individual flavonoids from encapsulated OFC extracts, IG2 was the most efficiently encapsulated (from 55.2 ± 4.2 to 75.3 ± 0.9%) using the system TW2. These results can also be compared with those reported by Fernández-Repetto et al. [27], who reported slightly higher Ees% for IG2 in the range of 63.7 ± 2.3 to 71.8 ± 1.

Overall, the most efficient double emulsion system was TW2, as it showed the better results for total betalains, total phenolic acids, and total flavonoids, as well as among individual compounds for indicaxanthin, vulgaxanthin III, vulgaxanthin I, piscidic acid, quercetin glycoside 2 (QG2), isorhamnetin glucoxyl-rhamnosyl-rhamnoside-2 (IG2), and quercetin-3-rutinoside (rutin) (Table 5). This means that this system would be the most suitable to protect indicaxanthin and other bioactives from *Opuntia ficus-indica* var. *Colorada* pulps from degradation by different environmental and intrinsic factors.

Variations in the individual and total encapsulation efficiency of the different compounds present in OFC extracts, as well as in the different formulations, could be attributed to their composition, the solubility of each individual compound into the emulsions, and the shear stress conditions applied in each system.

In the case of TW emulsions, betalains were the compounds with the highest encapsulation efficiency values, which fits with their water-soluble conformation; however, in SC emulsions no significant differences were observed between the different families of compounds. It could be expected that those compounds with higher water solubility would be the ones that encapsulate more effectively, but the results suggest that the less hydrophilic compounds were also included in the systems, which is why water-in-oil-in-water emulsions are suitable for the encapsulation of both hydrophilic and non-hydrophilic compounds [12]. In addition, although both systems contain PGPR as the lypophilic emulsifier, it appears that the presence of Tween 20 as a hydrophilic emulsifier in comparison with sodium caseinate might play a crucial role in achieving superior efficiencies for the TW system. This could be attributed to the water retention capacity of sodium caseinate [30], which leads to an increase in the viscosity of emulsions and, therefore, variations in their texture, consistency, and homogeneity. In addition, even in small quantities, the presence of guar gum, arabic gum, and gelatin in its composition, which are all considered thickening and gelling agents, could also be contributing to these variations.

#### 3.3.2. Morphology of Double Emulsion Systems (W_1_/O/W_2_)

**Figure 2:**
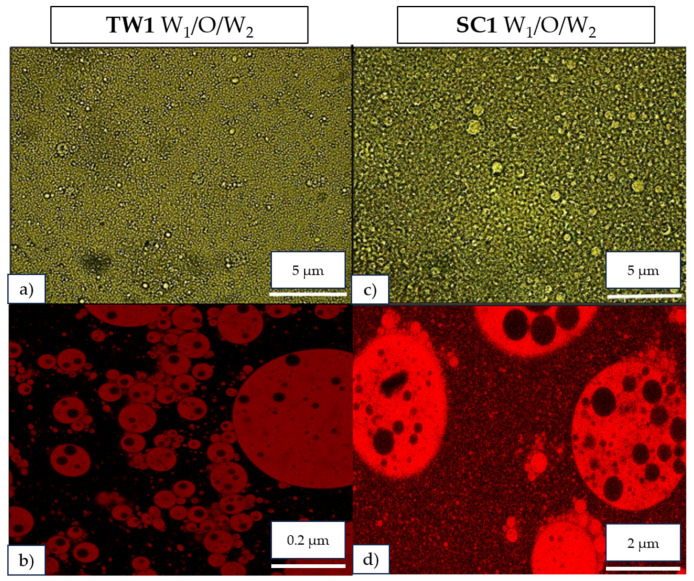
Optical and confocal microscopy images of double emulsion systems (W_1_/O/W_2_) TW1 (images (**a**,**b**)) and SC1 (images (**c**,**d**)) with encapsulated *O. ficus-indica* var. *Colorada* pulp (OFC) extracts.

Optical and confocal microscopy observations revealed that all formulated double emulsions were correctly formed, so it is possible to recognize their different layers (Figure 2). As expected, the emulsions were formed by an inner phase of small droplets (W_1_) surrounded by the oil phase (O), giving place to the inner emulsion (W_1_/O). In addition, these primary emulsion particles were covered by the outer continuous phase (W_2_), thereby achieving the formation of the complete double emulsion systems (W_1_/O/W_2_). The small, colored droplets in the W_1_ phase also confirm the introduction of OFC extracts into the inner phase. Specifically, confocal microscopy using the Nile Red dye allowed us to stain the oil droplets from the inner emulsion phases as seen in images (b) and (d). Optical and confocal microscopy images of the double emulsion systems TW1 and SC1 (both containing 1 g of OFC pulp extract encapsulated) can be seen in Figure 2.

With respect to the shape of the systems and considering the scale of the images (Figure 2), the double emulsions formulated with Tween 20 (TW) as the emulsifier showed smaller droplets than those elaborated with sodium caseinate (SC). Similar microscopy images were reported by Sanhueza et al. [31] in a study of the encapsulation of pomegranate peel extracts by double emulsion systems, using polyglycerol polyricinoleate as the emulsifier. The size of the particles could be used to make a prediction of the stability of the system, where smaller particles can retard the coalescence and breakage of the emulsion droplets [12]. In order to confirm it, particle size and zeta potential (both predictors of stability in dispersions) were analyzed in the following sections.

#### 3.3.3. Physical Stability of Double Emulsions

**Figure 3:**
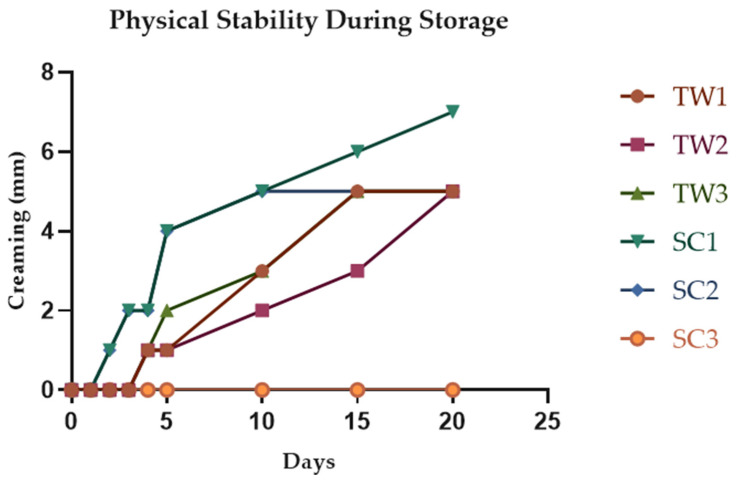
Evolution of the physical stability (creaming) of the double emulsions based on Tween 20 (TW) and sodium caseinate (SC) with encapsulated *O. ficus-indica* var. *Colorada* pulp extracts during storage at 7 °C for 20 days.

An emulsion is considered unstable when the dispersed phase and the continuous phase are separated. Phase separation can be determined visually by the formation of visible creaming [31]. The two types of double emulsions prepared in the present study (TW and SC) showed a yellow-orange color, due to the presence of encapsulated betaxanthins (mainly indicaxanthin) from OFC pulp extracts. Double emulsions elaborated with Tween 20 (TW1, TW2, TW3) started their creaming on day 4 (1 mm) of storage at 7 °C, increasing to 5 mm up to day 20 (Figure 3). Regarding, the double emulsions prepared with sodium caseinate (SC1, SC2, SC3), SC1 and SC2 showed a slight phase separation from day 2 (1 mm) that increased through cold conservation up to 7 mm in SC1 and 5 mm in SC2 on day 20. In contrast, SC3 did not undergo phase separation at any time during the 20 days of conservation at 7 °C, but the presence of small bubbles interfered in the particle size and Z potential measurements (Figure S3, Supplementary Materials).

In general, the obtained results indicated that stability during storage at 7 °C of the emulsions formulated with Tween 20 was significantly higher than that observed for those made with caseinate (Figure S3), except for SC3, which did not show any phase separation due to its high consistency. These results are consistent with the data published by Parralejo-Sanz et al. [23], who reported that formulations with Tween 20 were more stable during cold storage than those elaborated with sodium caseinate when they were used to encapsulated *Opuntia stricta* var. *dillenii* extracts rich in betanin. In the present work, in the TW double emulsions the creaming was formed in the upper area, and in the SC double emulsions it appeared in the lower region (Figure S3, Supplementary Materials).

#### 3.3.4. Particle Size and Zeta Potential

Emulsion systems tend to be more stable when they show small particle sizes in the dispersed phase (W_1_) as it helps to avoid unions between them (creaming, coalescence, or flocculation) and the subsequent disintegration of the emulsion [32]. Furthermore, emulsions with smaller particles tend to have a more fluid-like flow behavior, whereas emulsions with larger particles can become more viscous and show a pseudo-plastic flow behavior.

**Figure 4:**
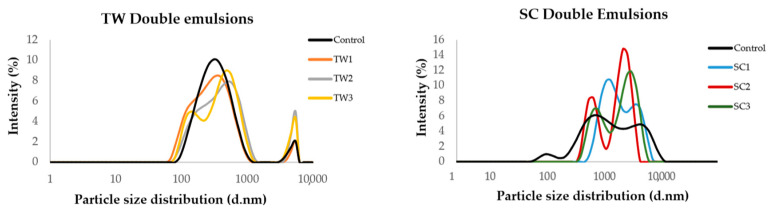
Particle size distribution (nm) of TW and SC double emulsion systems without (control) and with encapsulated extracts from *O. ficus-indica* var. *Colorada* pulps on Day 0 of elaboration.

In Figure 4, we can see the particle size distribution analysis of the TW and SC double emulsion systems just elaborated (day 0). Regarding TW emulsions, all of them showed two particle size populations, with the most important being the first one with an average size of 280 nm. The control emulsion showed more homogeneity in the particle size distribution, while those with encapsulated OFC extract were more heterogeneous. With respect to SC emulsions, the control emulsion showed three particle size populations, while the emulsions with encapsulated OFC extract showed two (Figure 4). In this case, all of the samples showed more heterogeneity in comparison with TW emulsions, which is attributable to the composition of these systems.

The polydispersity index (PDI) describes the width or spread between the particles of a dispersion attending to their molecular size. The PDI can range from 0 to 1; when it is below 0.1, it indicates a colloidal system with particles of relatively uniform size, while values exceeding 0.1 suggest a broader range of particle sizes, reflecting a polydisperse distribution [33]. The TW control emulsion showed values of 0.2 ± 0.03 PDI, while for TW emulsions with OFC extract encapsulated, the values obtained were 0.37 ± 0.01, 0.4 ± 0.1, and 0.4 ± 0.0 for TW1, TW2, and TW3, respectively. Regarding SC emulsions, PDI values of 0.4 ± 0.1 were obtained for the control, while for SC emulsions PDIs were 0.5 ± 0.04 and 0.52 ± 0.61 ± 0.08. In both TW and SC emulsions, PDI results defined that the dispersions presented heterogeneous populations, more notably for SC emulsions. These results can be compared with those reported by Mohammed et al. [34], who encapsulated beetroot extracts abundant in betalains by micro- and nanoemulsion polydisperse systems, obtaining PDIs of 0.34 ± 0.1 and 0.5 ± 0.03, respectively.

**Figure 5:**
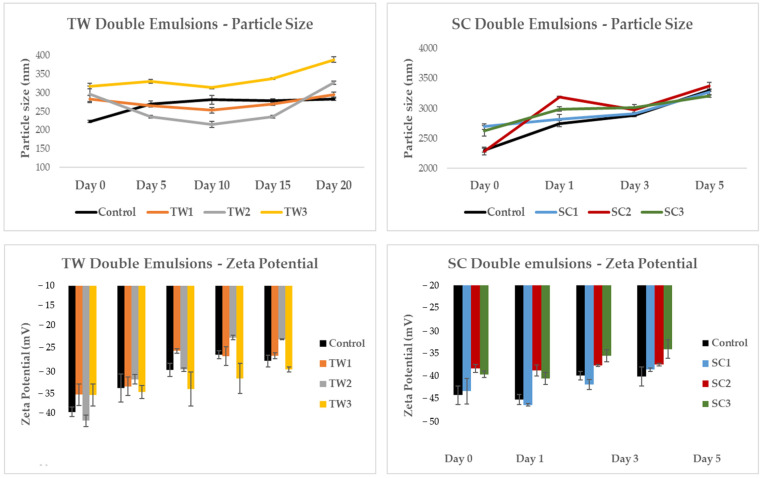
Particle size and zeta potential data from TW and SC emulsions with OFC extract en-capsulated and without (control) during 20 day storage at 7 °C.

In Figure 5 we can see the individual data of the particle size and zeta potential of each emulsion during conservation. TW emulsions showed smaller particle sizes than SC emulsions, specifically, the particles that constituted SC emulsions showed ten times larger sizes (from 2286 ± 42 to 3373 ± 64 nm) than TW emulsions (from 223 ± 3 to 389 ± 7 nm). Velderrain-Rodríguez et al. [30] used different surfactants to encapsulate phenolic compounds extracted from mango peels, and they also obtained lower particle sizes when using Tween 20. With respect to the different particle sizes in both types of double emulsions with encapsulated OFC pulp extracts at different concentrations (1, 2, or 3 g), those with higher concentrations (3 g of OFC extract) showed the biggest droplet sizes (from 314 ± 3 to 389 ± 7 for TW3 and from 2622 ± 86 to 3202 ± 21 for SC3), while those with no extract encapsulated (control emulsions) showed the smallest ones (from 223 ± 3 to 283 ± 3 for TW control and from 2301 ± 34 to 3001 ± 19 for SC control) (Figure 5).

Attending to changes during storage at 7 °C, an increase in all of the systems was observed, especially in SC double emulsions, which showed the most evident changes in particle size distribution (Figure 3 and Figure 5). In fact, from day 5 onwards, the particle size and zeta potential data of the SC emulsion could not be measured due to the gelatinous consistency of the emulsion, which made the analysis difficult. Robert et al. [17] also reported an increase in the droplet size of their emulsions made with polyglycerol poliricinoleate when they encapsulated *Opuntia ficus-indica* extracts and stored them for 28 days at 4 °C, reporting particle size values from 2560 ± 210 nm on day 0 to 3830 ± 120 nm on day 28.

On the other hand, zeta potential measures the electrostatic repulsion or attraction between particles in suspension; this value indicates the potential difference at the phase boundary and provides information on dispersion, aggregation, and flocculation processes in emulsions [35]. The critical absolute value of a zeta potential above which an emulsion can be considered stable is 30 mV because it means that there is significant electrostatic repulsion between the dispersed particles. This repulsion helps to prevent droplet coalescence and thus contributes to the stability of the emulsion. Equally charged particles repel each other, so those with high charges will resist flocculation and aggregation for longer periods of time [36].

With respect to zeta potential (Figure 5), the values obtained on day 0 (double emulsions just elaborated) ranged from ∣34.7∣ ± 2.4 to ∣40.6∣ ± 1.3 mV in Tween 20 (TW) emulsions and from ∣38.2∣ ± 0.9 to ∣43.2∣ ± 4.8 mV in sodium caseinate (SC) emulsions. These results could ensure the initial stability of both systems. Ribeiro et al. [37] produced O/W emulsions with hydroglycolic extracts of *Opuntia ficus-indica* and reported zeta potential values of ∣42.9∣ ± 0.9 after 24 h.

Zeta potential values of TW double emulsions (based on Tween 20) decreased throughout the storage time. This fact seemed to be related to the beginning of the emulsion creaming described above (Figure 3). From the tenth day of conservation, TW1 and TW3 systems (containing 1 and 3 g of OFC pulp green extract, respectively) began to turn more unstable showing Z potential values of ∣24.8∣ ± 0.7–∣29∣ ± 0.1 mV, respectively, while the TW2 system (containing 2 g of OFC pulp green extract) remained stable, and it showed values above ∣30∣ mV until day 20 of storage. In the case of SC emulsions (based on sodium caseinate), as already mentioned, from day 5 no more measurements could be performed due to its hard consistency (Figure 5). Nevertheless, the trend of the zeta potentials in SC emulsions (from ∣35.5∣ ± 1.3 to ∣38.5∣ ± 0.4 mV on the third day of measurement) could indicate the emulsion stability for longer periods than TW emulsions. However, as they could not be fully measured, their stability cannot be assumed with these parameters (Figure 5).

Medina-Pérez et al. [38] reported that the higher the concentration of the internal aqueous phase is (W_1_) the more unstable the emulsion system can turn, as it will cause a propensity of the droplets to bind together and form larger droplets, but, in the case of the present study, the analysis of zeta potential did not show statistically significant differences between the double emulsions with distinct OFC extract concentrations. Overall, the particle sizes of the double emulsion systems based on Tween 20 would be predicted to be more stable as they showed smaller values and smaller increases during storage, which were especially observed for TW2 (containing 2 g of OFC pulp green extract) (Figure 4 and Figure 5). These results are in concordance with the data already mentioned for the physical stability (creaming) of the double emulsions (Figure 3). With regard to zeta potential, the double emulsion systems based on sodium caseinate showed higher values, but as not all their measurements could be performed, a full prediction of their stability during storage cannot be made.

### 3.4. In Vitro Gastro-Intestinal Digestion Assay

Double emulsions with encapsulated OFC pulp extracts were subjected to in vitro gastro-intestinal digestion following the INFOGEST© protocol in order to evaluate the effect of the extract encapsulation on the stability and bioaccessibility of the main individual bioactive compounds (betalains and phenolic compounds) present in OFC extract.

#### 3.4.1. Confocal Laser Microscopy Analysis of Gastro-Intestinal Digestion Phases

**Figure 6:**
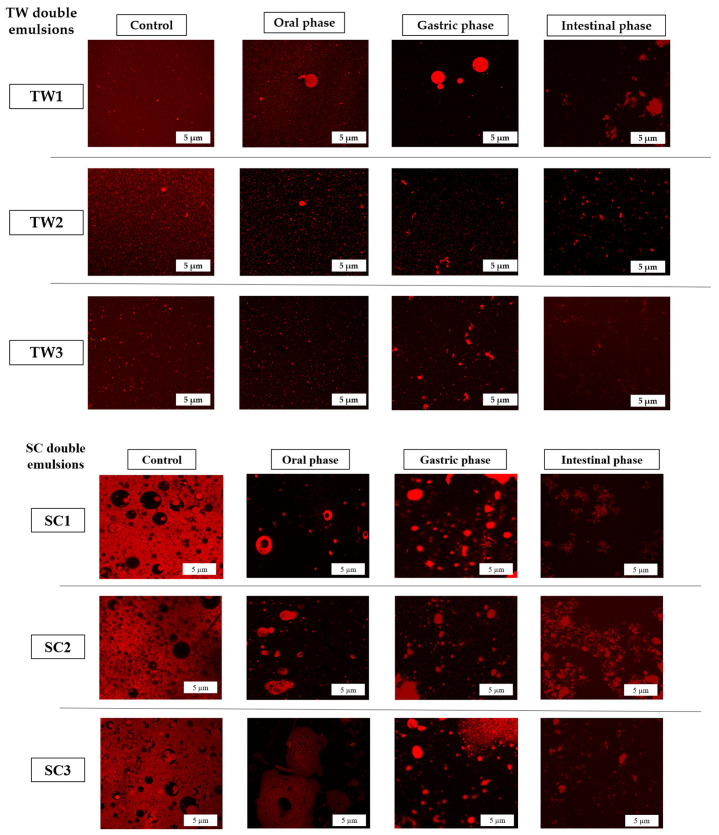
Confocal microscopy images of the double emulsion systems TW and SC with encapsulated *O. ficus-indica* var. *Colorada* pulp extract during in vitro gastro-intestinal digestion: control (refers to the non-digested systems), oral phases, gastric phases, and intestinal phases.

Confocal Laser Microscopy allows us to obtain detailed information on complex structures such as the double emulsions [39], as was mentioned before. Figure 6 shows the changes in the structure of TW and SC double emulsion systems with encapsulated OFC extract during in vitro gastro-intestinal digestion phases.

Control TW and SC double emulsion systems with encapsulated OFC extracts (non-digested emulsions) and emulsions in the oral phase seemed to remain intact (Figure 6). In contrast, at the gastric phase only some particles of the double emulsions were still complete, while others had already begun their disintegration process. When the intestinal phase was reached, all particles of the double emulsions had been decomposed (Figure 6). Similar results were reported by Hu et al. [40], who co-encapsulated epigallocatechin-3-gallate and quercetin in double emulsion hydrogel beads and exposed them to in vitro gastro-intestinal digestion, showing that the digestion phases of the gastro-intestinal tract caused the breakdown of the particle structures. This can be attributed to the action of the digestive fluids and enzymes, which evoke stronger reactions at the gastric and intestinal stages (proteases, lipase, bile salts, hydrochloric acid, and so on). Kaimainen et al. [15] analyzed the digestion of double emulsions with encapsulated betalains and reported that, in the intestinal phase, the emulsion particles experienced the loss of their original structure, which caused the release of the bioactive compounds. In this way, in the present work, after the digestion of TW and SC double emulsion systems, the encapsulated betalains and the phenolic compounds in them were theoretically released and free to be absorbed by the intestinal epithelium.

#### 3.4.2. Particle Size and Zeta Potential of Double Emulsions during Gastro-Intestinal Digestion

Particle size and zeta potential of double emulsions based on Tween 20 (TW) and based on sodium caseinate (SC) with encapsulated OFC pulp extract throughout in vitro gastro-intestinal digestion were analyzed in each digestion phase to determine the stability of the emulsions along the process. These data are presented in Table 6.

With respect to the double emulsion systems based on Tween 20 (TW), smaller particle sizes were observed in the oral phase (217 ± 3–222 ± 9 nm), followed by the gastric phase (225 ± 4–276 ± 6 nm). It was after the intestinal phase that the most important increase in particle size was observed (1387 ± 84–2374 ± 117 nm). This fact could be attributed to the action of the digestive enzymes and fluids, which cause the coalescence, aggregation, and phase separation of the emulsion particles while the digestion process advances. However, theoretically, in the gastric phase some of the double emulsion particles must be already beginning to degrade (Figure 6). Nevertheless, the obtained zeta potential values at this gastric phase (values from ∣42.1∣ ± 2.8 to ∣48.7∣ ± 0.6 mV) indicate an apparent stability. After that, when the intestinal phase was reached an important decrease in zeta potential was observed (values from ∣11.7∣ ± 0.5 to ∣13.2∣ ± 0.7 mV) for these emulsions which seemed to be an indicator of the breakage of the emulsion particles.

For its part, double emulsions based on sodium caseinate (SC) showed an increase in particle size values from the oral phase (1343 ± 165–1879 ± 25 nm) to the gastric phase (2254 ± 197–2697 ± 64 nm). However, a significant reduction in particle size was observed in the intestinal phase (1287 ± 176–1660 ± 124 nm) for this formulation. With respect to the zeta potential of SC systems, a high variation was observed in the oral and gastric phases. At the gastric phase, the obtained potential size values indicated an instability of the system (values from ∣14.8∣ ± 2.9 to ∣24.2∣ ± 4.5 mV) that were attributed to the process of particle disintegration by the action of gastric lipase already present at this digestion phase. Regarding the intestinal phase, the zeta potential values (from ∣14.8∣ ± 0.5 to ∣18.4∣ ± 0.3 mV) also indicated instability of the emulsion systems, meaning the breakage of the emulsion particles. This is also observed in Figure 6.

The particle sizes and zeta potentials of both TW and SC double emulsions with encapsulated OFC extract (Table 6) showed similar values at the end of digestion (intestinal phase). The same event was reported by Parralejo-Sanz et al. [23] when they encapsulated *Opuntia stricta* var. *dillenii* extracts also using Tween 20 and sodium caseinate as emulsifiers. Velderraín-Rodríguez et al. [41] also analyzed the changes in particle size during in vitro gastro-intestinal digestion in their double emulsion in which red beet extract was encapsulated, and they also reported the formation of larger droplets as the digestion process progressed, achieving the biggest sizes at the end of it. Han et al. [42] co-encapsulated quercetin and insulin by double emulsions using different emulsifiers and submitted them to in vitro digestion. They reported a decrease in absolute zeta potential values by comparing the beginning of the process with the end of the intestinal phase, similarly to what was observed in the present work.

#### 3.4.3. Digestive Stability, Recovery, and Bioaccessibility of Encapsulated OFC Bioactives by Double Emulsions

**Figure 7:**
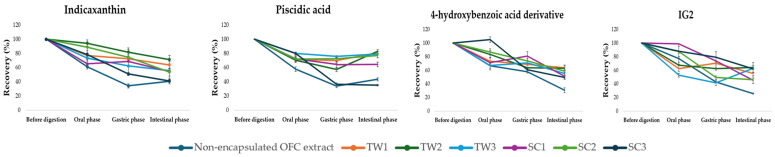
Recovery (%) of the main compounds encapsulated in the double emulsions based on Tween 20 (TW) and sodium caseinate (SC) from *Opuntia ficus-indica* var. *Colorada* pulps’ extracts after in vitro gastro-intestinal digestion (based on the results obtained for digestive stability).

Digestive stability and bioaccessibility of the main bioactives from *Opuntia ficus-indica* var. *Colorada* pulp green extracts and the encapsulated ones by the double emulsion systems based on Tween 20 (TW) and on sodium caseinate (SC) are shown in Table 7 and Table 8, respectively. Recovery data of the individual bioactives through digestion process, calculated on the basis of the digestive stability and represented graphically, are shown in Figure 7.

The changes in the content of each individual bioactive compound of OFC extract and from the encapsulated OFC extract by double emulsions during the oral, gastric, and intestinal phases were analyzed. The values of digestive stability, recovery, and bioaccessibility obtained for the individual bioactives of non-encapsulated OFC pulp extract were significantly lower than those obtained for the encapsulated OFC extract by double emulsions. These results suggest that the double emulsion systems in the present work may achieve an adequate protection of these bioactives during gastro-intestinal digestion. Possibly, the structure of the double emulsions creates a physical barrier around the bioactive compounds, preventing or diminishing their contact with enzymes and digestive solutions, thus reducing the rate of digestion. This would allow for the controlled release of the compounds throughout the process.

Overall, the most abrupt changes in the quantity of individual bioactive compounds content were observed after the gastric phase (Table 7 and Table 8, Figure 7). This fact can be logical when the action of gastric fluids and enzymes affecting the stability of the OFC bioactives was taken into account. Montiel-Sánchez et al. [10] reported the same events when they submitted *Myrtillocactus geometrizans* extracts, also abundant in betalains and phenolic compounds, to the in vitro gastro-intestinal digestion protocol.

With respect to betalain compounds, only indicaxanthin was detected and quantified by HPLC analysis in all of the digestion phases of the gastro-intestinal digestion of the encapsulated OFC extracts by the two studied double emulsion systems. This fact could be explained by the higher indicaxanthin concentration in OFC extract (508 ± 10 µg/g d.w.) with respect to the content of other minor betalains (vulgaxanthins and portulacaxanthin). Attending to stability, the content of indicaxanthin in the non-encapsulated OFC pulp green extract during digestion ranged from 389 ± 3 µg/g d.w. in the oral phase to 323 ± 11 µg/g d.w. in the intestinal phase, while in the encapsulated OFC extracts by the TW2 emulsion system, which showed the best results, indicaxanthin’s content ranged from 802 ± 52 µg/emulsion in the oral phase to 700 ± 26 µg/emulsion in the intestinal phase (Table 7).

Indicaxanthin’s bioaccessibility was 82.3 ± 6% for TW2 (Table 8). As already mentioned, bioaccessibility is a very important parameter when the main interest is the compounds’ bioactivity and its bioavailability, so, in fact, TW2 system could be the most interesting one when trying to protect indicaxanthin and thus maintain its biological interest. Fernández-Repetto et al. [27] encapsulated extracts of a purple variety of *Opuntia ficus-indica* with different hydrocolloid excipients and reported indicaxanthin bioaccessibilities from 78.7 ± 3.6 to 98.9 ± 1.6%. The differences observed among the indicaxanthin bioaccessibilities in the present work with respect to the data reported in the bibliography could be related to the different composition of the encapsulation formulations, the composition of bioactives of the different *Opuntia* fruits, and the different processes used to encapsulate them.

Regarding phenolic compounds, piscidic acid, 4-HAD, and IG2 were the phenolic compounds found by HPLC analysis through the digestion phases of the double emulsion systems with encapsulated OFC extracts.

Piscidic acid showed the highest content in OFC extracts out of all the phenolic compounds. The TW2 double emulsion showed the best stability values in a range between 3056 ± 290 µg/emulsion in the oral phase and 3765 ± 198 in the intestinal phase µg/emulsion, where we can see that stability was higher in the intestinal phase than in the oral phase, meaning a high protection of this compound with the double emulsion. In contrast, piscidic acid stability in the non-encapsulated OFC pulp green extracts had values ranging from 1112 ± 116 µg/g d.w. in the oral phase and 586 ± 79 µg/g d.w. in the intestinal phase (Table 7). With respect to piscidic acid’s bioaccessibility, the double emulsion system TW2 presented the highest values, being up to 82.8 ± 1.5% (Table 8). As an exception, only the double emulsion system SC3 (based on sodium caseinate, containing 3 g of OFC pulp green extract) showed a lower bioaccessility value for piscidic acid (35.2 ± 3.9%), in comparison with the non-encapsulated extract (43.6 ± 2.2%).

Among individual flavonoids present in OFC extracts, IG2 stability values in non-encapsulated OFC extract ranged from 24.6 ± 2.6 µg/g d.w. in the oral phase to 8.3 ± 0.3 µg/g d.w. in the intestinal phase. Regarding double emulsions, TW2 again showed the best results, with values between 28 ± 1.1 µg/emulsion in the oral phase and 16 ± 1.9 µg/emulsion (Table 7). Fernández-Repetto et al. [27] reported bioaccessibilities of 62.4 ± 0.5% for IG2, when they encapsulated purple *Opuntia ficus-indica* extracts with different hydrocolloid excipients, so, in this case, even though a different variety of *Opuntia ficus-indica* was used, their bioaccessibilities showed similar results. The results obtained indicate that both the TW and SC emulsion systems remain promising in enhancing the protection of the bioactive compounds found in OFC pulp extracts from the digestion process, as evidenced by their stability, recovery, and bioaccessibility. In comparison with the non-encapsulated extract. This suggests that most of the encapsulated compounds could potentially be absorbed in the digestive tract, leading to increased bioavailability and an increased efficacy in exerting their intended biological functions in the body’s target tissues. It should be noted that the TW2 double emulsion system, containing Tween 20 as an emulsifier and 2 g of OFC pulp green extract, emerged as particularly effective in protecting bioactives during digestion as it displayed superior performance in increasing the bioaccessibility of indicaxanthin, piscidic acid, and IG2.

## 4. Conclusions

For the first time, *Opuntia ficus-indica* var. *Colorada* (a Spanish cactus fruit variety from the Canary Islands with orangish colorations) pulp extracts abundant in betalains (mainly indicaxanthin) and phenolic compounds (mainly phenolic acids and flavonoids) have been encapsulated by double emulsion water-in-oil-in-water systems with the aim of increasing their physical stability during storage, as there are multiple endogenous and exogenous factors that can affect their stability. The first double emulsion system was based on the hydrophilic surfactant Tween 20 (TW), while the second one was based on the protein emulsifier sodium caseinate (SC). Both were assayed in three different OFC pulp green extract concentrations (1, 2, and 3 g of OFC extract/total emulsion system). TW double emulsion systems showed smaller particle sizes (between 236 ± 4 and 389 ± 7 nm) than the SC double emulsion systems (between 2286 ± 64 and 3373 ± 64 nm) and, even though zeta potential absolute values were higher for SC (from ∣35.5 ∣ ± 1.3 to ∣46.2∣ ± 0.3 mV) than for TW (from ∣21.8∣ ± 0.6 to ∣40.6∣ ± 1.3 mV), the 20 day storage at 7 °C creaming analysis revealed that TW double emulsion systems were more stable. Moreover, TW double emulsion systems showed higher encapsulation efficiencies than SC double emulsions, especially with TW2 (which contained 2 g of OFC pulp green extracts), reaching an encapsulation efficiency of 97.2 ± 0.4% for indicaxanthin and of 97.3 ± 2.7% for piscidic acid.

Digestive stability and bioaccessibility of the individual betalains and phenolic compounds from OFC pulp extracts were also evaluated by the standardized INFOGEST protocol, in comparison with the non-encapsulated extracts. The latter presented lower digestive stability, recovery, and bioaccessibility in comparison with the bioactives encapsulated. In particular, the best results were obtained for the double emulsion system TW2, with an indicaxanthin stability of 802 ± 52 μg/emulsion in the oral phase and of 700 ± 26 μg/emulsion in the intestinal phase and a bioaccessibility of 82.3 ± 6%.

Overall, both TW and SC double emulsion systems were apparently efficient in the protection of indicaxanthin and the other bioactives present in *Opuntia ficus-indica* var. *Colorada* pulp green extracts. The ability to encapsulate bioactive compounds in double emulsions opens up new possibilities for innovative product development in the pharmaceutical and food industries. This includes the creation of functional foods enriched with bioactive nutrients with a controlled release and enhanced stability, as well as the formulation of more effective and safer medicines. Therefore, the proposed encapsulation double emulsion systems, especially TW2, could be a promising strategy for the elaboration of bioactive functional ingredients abundant in the natural dye indicaxanthin and phenolic compounds of proven biological interest.

## Figures and Tables

**Table 1:** Physico-chemical characteristics of prickly pear fruits of *Opuntia ficus-indica* var. *Colorada*.

*Opuntia ficus-indica* var. *Colorada* ^1^	
Pulp colour	Orange
Weight (g)	122 ± 20
Apical caliber (cm)	6.8 ± 0.9
Equatorial caliber (cm)	5.1 ± 0.2
Moisture (%)	82.5 ± 1.3
pH	6.5 ± 0.1
Soluble solids (°Brix)	14.8 ± 0.1
Titratable acidity (%)	0.01 ± 0.0
Pulp colour (CIELAB)	
L*	40.2 ± 3.9
a*	10.8 ± 2.5
b*	20.6 ± 2.4

^1^ Analysis were conducted in triplicate (n = 3).

**Table 2:** Composition of the double emulsion systems (W_1_/O/W_2_) to encapsulate green extracts of *O. ficus-indica* var. *Colorada* fruit pulps.

TW Double Emulsion	SC Double Emulsion
Primary water phase (W_1_)	
2.2 g of NaCl 0.1 M 0.3 g of glycerol OFC pulp extract (1 g on TW1, 2 g on TW2, 3 g on TW3)	2.25 g TRIS buffer with 6% gelatin OFC pulp extract (1 g on SC1, 2 g on SC2, 3 g on SC3)
Oil phase (O)	
7 g of MCT oil 0.5 g of PGPR	5.2 g of MCT oil 0.4 g of phosphatidylcholine 1.4 g of PGPR
Secondary water phase (W_2_)	
29.4 g of NaCl 0.1 M 0.6 g of Tween 20	17.04 g of 3% sodium caseinate 3.91 g of glycerol with 10% of NaCl 0.0525 g of guar gum 0.0795 g of arabic gum

**Table 3:** Characterization of the total betalains and phenolic compounds detected in *Opuntia ficus-indica* var. *Colorada* pulps analyzed by HPLC-DAD and HPLC-MS/MS ^1^.

Peak ^2^	Compound	Rt (Min)	λ Max (nm)	[M + H]^+^	[M + H]^−^	MS/MS (*m*/*z*)
1	Portulacaxanthin III	3.28	471	269.11		225.14, 136.06
2	Vulgaxanthin III	3.78	474	326.14		325.14, 307.13, 220.10
3	Vulgaxanthin I	4.15	470	340.11		308.09, 116.07, 84.04, 76.02
4	Vulgaxanthin II	5.48	474	341.10		292.20, 147.04, 72.08
5	Indicaxanthin	8.67	478	309.11		263.10, 217.10, 70.06
6	Piscidic acid	10.41	232, 275	257.07		191.07, 147.04, 119.05, 107.05
7	Betanin	10.96	534	551.15		390.10, 389.10
8	4-hydroxybenzoic acid derivative (4-HAD)	33.76	270		299	137, 119, 93
9	Quercetin glycoside I (QG1)	38.2	266, 351	426.24		303.05, 191.07, 120.08
10	Quercetin glycoside II (QG2)	39.34	269, 350	653.28		303.05, 177.05
11	Isorhamnetin glucoxyl-rhamnosyl-rhamnoside (IG1)	40.07	254, 354	771.23		625.18, 317.07, 85.03
12	Isorhamnetin glucosyl-rhamnosyl-pentoside (IG2)	41.39	253, 354	757.22		317.07, 167.07, 86.10
13	Quercetin-3-rutinoside (Rutin)	42.16	250, 343	611.23		303.05, 229.11, 137.07

^1^ Data were confirmed by comparison with those previously published by Gómez-Maqueo et al. [3]. ^2^ Peak numbers are according to Figure S1 (Supplementary Materials).

**Table 4:** Content in the main betalains and phenolic compounds of green extracts from *Opuntia ficus-indica* var. *Colorada* prickly pear fruit pulps and encapsulated extract by double emulsion systems TW and SC.

Compound	Content (µg of Compound/Emulsion)						
	OFC Pulp Extract	TW Double Emulsions ^1^			SC Double Emulsions ^2^		
		TW1 ^3^	TW2 ^3^	TW3 ^3^	SC1 ^3^	SC2 ^3^	SC3 ^3^
Portulacaxanthin III (Bx-glycine)	26.8 ± 1.3 ^b^	20.9 ± 4.8 ^b^	32.5 ± 6.1 ^b^	58.6 ± 1.6 ^c^	15.4 ± 0.7 ^a^	31.7 ± 4.3 ^b^	51.2 ± 5.5 ^c^
Vulgaxanthin III (Bx-asparagine)	14.6 ± 3.4 ^a^	12.6 ± 2.4 ^a^	26.4 ± 3.8 ^b^	39.8 ± 6.6 ^c^	9.8 ± 0.3 ^a^	23.3 ± 5.5 ^b^	39.3 ± 3.1 ^c^
Vulgaxanthin I (Bx-glutamine)	13.7 ± 3 ^a^	11.6 ± 1.1 ^a^	25.2 ± 4.4 ^b^	36.3 ± 1.3 ^c^	11.5 ± 1.7 ^a^	23.4 ± 3.5 ^b^	35.6 ± 2.7 ^b^
Vulgaxanthin II (Bx-glutamic acid)	18.8 ± 2.7 ^a^	16.3 ± 3.1 ^a^	28.6 ± 0.7 ^b^	35.4 ± 3.5 ^c^	12.8 ± 0.6 ^a^	21.7 ± 8.8 ^b^	36.3 ± 6.4 ^c^
Indicaxanthin (Bx-proline)	508 ± 10 ^a^	243 ± 34 ^a^	850 ± 40 ^b^	1366 ± 16 ^c^	257 ± 20 ^a^	670 ± 71 ^b^	1444 ± 170 ^c^
Piscidic acid	2641 ± 92 ^a^	1694 ± 75 ^a^	4753 ± 210 ^b^	11,449 ± 156 ^d^	1614 ± 176 ^a^	6248 ± 356 ^c^	10,345 ± 436 ^d^
4-hydroxybenzoicacid derivative (4-HAD)	117 ± 6 ^a^	62.4 ± 2 ^a^	295 ± 9 ^b^	471 ± 16 ^c^	62.9 ± 2 ^a^	214 ± 57 ^b^	285 ± 30 ^b^
Quercetin glycoside 2 (QG2)	18.1 ± 1.4 ^a^	14.8 ± 1.4 ^a^	35.5 ± 1.6 ^b^	43 ± 4.7 ^c^	12.3 ± 4 ^a^	27.1 ± 4.2 ^b^	43.6 ± 0.6 ^c^
Isorhamnetin glucoxyl-rhamnosyl-pentoside (IG2)	31.9 ± 1.4 ^a^	28.9 ± 4.2 ^a^	41.1 ± 1.4 ^a^	50.8 ± 7.1 ^a^	17.6 ± 1.5 ^a^	40.3 ± 2.8 ^a^	60.4 ± 1.2 ^b^
Quercetin-3-rutinoside (Rutin)	16.2 ± 0.1 ^a^	10.1 ± 0.1 ^a^	21.9 ± 1 ^b^	43.1 ± 2.4 ^c^	13 ± 1.3 ^a^	28.6 ± 2 ^b^	33.2 ± 2.4 ^b^
Total Betalains	382 ± 20.4 ^a^	304 ± 45 ^a^	962 ± 55 ^b^	1536 ± 26 ^c^	306 ± 23 ^a^	770 ± 93 ^b^	1606 ± 189 ^c^
Total Phenolic Acids	3758 ± 98 ^a^	2766 ± 96 ^a^	6048 ± 219 ^b^	12,920 ± 113 ^c^	2676 ± 178 ^a^	7462 ± 413 ^b^	12,630 ± 466 ^c^
Total Flavonoids	66.2 ± 2.9 ^a^	44.8 ± 5.7 ^a^	98.5 ± 4 ^b^	137 ± 14 ^c^	43 ± 6.8 ^a^	96 ± 9 ^b^	137 ± 4 ^c^

Superscript letters indicate statistically significant differences (*p* ≤ 0.05) between the different samples of the double emulsion systems for each individual compound and for the total of each family of compounds. Analyses were conducted in triplicate (n = 3). ^1^ TW: double emulsion systems based on Tween 20; ^2^ SC: double emulsion system based on sodium caseinate. ^3^ Content of *Opuntia ficus-indica* fruit pulp green extract in the double emulsions as follows: (1) 1 g of OFC pulp green extract; (2) 2 g of OFC pulp extract; and (3) 3 g of OFC pulp green extract.

**Table 5:** Encapsulation efficiency (%) of the main individual betalains and phenolic compounds from *O. ficus-indica* var. *Colorada* fruit pulp green extracts encapsulated by the double emulsion systems (W_1_/O/W_2_) TW and SC.

Compound	Encapsulation Efficiency (%)					
	TW Double Emulsions ^1^			SC Double Emulsions ^2^		
	TW1 ^3^	TW2 ^3^	TW3 ^3^	SC1 ^3^	SC2 ^3^	SC3 ^3^
Portulacaxanthin III	78.2 ± 4.7 ^b^	67.9 ± 8.3 ^b^	48.6 ± 0.8 ^b^	57.6 ± 1 ^b^	44.5 ± 1.6 ^a^	50.7 ± 8.7 ^b^
Vulgaxanthin III	86.6 ± 6.5 ^b^	90.4 ± 9.8 ^b^	74.4 ± 1.3 ^b^	67.6 ± 1.5 ^b^	59.8 ± 2.9 ^a^	53.3 ± 2.1 ^a^
Vulgaxanthin I	85 ± 7 ^b^	96.8 ± 1.8 ^b^	95.3 ± 6.2 ^b^	84 ± 0.9 ^b^	79.2 ± 3 ^b^	55.0 ± 4.3 ^a^
Vulgaxanthin II	86.5 ± 2.9 ^a^	66.5 ± 2.3 ^a^	64.8 ± 9.7 ^a^	68.3 ± 3.1 ^a^	92.4 ± 9.6 ^a^	82.7 ± 0.5 ^a^
Indicaxanthin	78.9 ± 2.5 ^a^	97.2 ± 0.4 ^a^	86.6 ± 2.6 ^a^	83.5 ± 8.1 ^a^	84.7 ± 1.1 ^a^	77.7 ± 5.1 ^a^
Piscidic acid	74 ± 5.1 ^a^	97.3 ± 2.7 ^a^	95.5 ± 6.3 ^a^	71.8 ± 6.4 ^a^	87.6 ± 4.7 ^a^	88.9 ± 1.5 ^a^
4-hydroxybenzoic acid derivative (4-HAD)	52.8 ± 2.4 ^b^	53.4 ± 2 ^b^	55.3 ± 3.4 ^b^	53.8 ± 1.6 ^b^	51.1 ± 4.9 ^a^	32.3 ± 2 ^a^
Quercetin glycoside 2 (QG2)	53 ± 8.6 ^a^	75.7 ± 2.9 ^a^	74.6 ± 7.1 ^a^	79.6 ± 4.3 ^a^	66.3 ± 5.0 ^a^	75.7 ± 10.3 ^b^
Isorhamnetin glucoxyl- rhamnosyl-pentoside 2 (IG2)	90.6 ± 1.3 ^b^	95.3 ± 0.9 ^b^	92.3 ± 8.3 ^b^	55.2 ± 4.2 ^a^	80.5 ± 5.2 ^b^	87.1 ± 1.6 ^a^
Quercetin-3-rutinoside (Rutin)	68 ± 9.3 ^a^	94.5 ± 4.6 ^a^	89.8 ± 1.4 ^a^	86.2 ± 7.5 ^a^	89.5 ± 10.2 ^a^	n.d. *
Total Betalains	83 ± 4.7 ^b^	84.4 ± 4.5 ^b^	73.9 ± 4.1 ^b^	62.8 ± 4.2 ^a^	72.1 ± 3.6 ^a^	63.9 ± 4.1 ^a^
Total Phenolic Acids	63.4 ± 3.8 ^a^	75.4 ± 2.4 ^a^	65 ± 5.3 ^a^	62.8 ± 3 ^a^	75.6 ± 3.9 ^a^	61.7 ± 3.7 ^a^
Total Flavonoids	70.5 ± 6.4 ^a^	71.4 ± 3.3 ^a^	67.4 ± 5.2 ^a^	73.7 ± 5.3 ^a^	69.1 ± 6.2 ^a^	81.4 ± 5.6 ^a^

Superscript letters indicate statistically significant differences (*p* ≤ 0.05) between the different samples of the double emulsion systems for each individual compound and for the total of each family of compounds. Analyses were conducted in triplicate (n = 3). ^1^ TW: double emulsion systems based on Tween 20; ^2^ SC: double emulsion system based on sodium caseinate. ^3^ Content of *Opuntia ficus-indica* fruit pulp green extract in the double emulsions as follows: (1) 1 g of OFC pulp green extract; (2) 2 g of OFC pulp extract; and (3) 3 g of OFC pulp green extract. * n.d.: not detected compound.

**Table 6:** Particle size (nm) and zeta potential (mV) values of TW and SC double emulsion systems with encapsulated extracts from *O. ficus-indica* var. *Colorada* pulps during in vitro gastro-intestinal digestion.

Digestion Phase	OFC Pulp Extract Content *	Particle Size		Zeta Potential	
		TW ^1^	SC ^2^	TW ^1^	SC ^2^
Control	1	252 ± 1 ^a^	2617 ± 168 ^b^	−32.4 ± 2.1 ^b^	−37.6 ± 5.1 ^a^
	2	202 ± 2 ^a^	3331 ± 35 ^b^	−34.7 ± 9.6 ^b^	−37.2 ± 1.6 ^a^
	3	206 ± 4 ^a^	2768 ± 140 ^b^	−31.9 ± 0.5 ^b^	−31.8 ± 2.3 ^b^
Oral	1	254 ± 4 ^a^	1832 ± 62 ^b^	−31.6 ± 0.9 ^b^	−24.1 ± 4.4 ^b^
	2	217 ± 3 ^a^	1879 ± 25 ^b^	−29.8 ± 0.8 ^b^	−25.2 ± 5 ^b^
	3	222 ± 9 ^a^	1343 ± 165 ^b^	−35.8 ± 1.2 ^b^	−31.7 ± 3.4 ^b^
Gastric	1	276 ± 6 ^a^	2697 ± 64 ^b^	−42.1 ± 2.8 ^a^	−24.2 ± 4.5 ^b^
	2	225 ± 4 ^a^	2419 ± 450 ^b^	−47.2 ± 2.1 ^a^	−14.8 ± 2.9 ^c^
	3	241 ± 7 ^a^	2254 ± 197 ^b^	−48.7 ± 0.6 ^a^	−17.9 ± 0.7 ^c^
Intestinal	1	1387 ± 84 ^b^	1660 ± 124 ^b^	−12.1 ± 0.6 ^c^	−15.8 ± 0.5 ^c^
	2	1787 ± 91 ^b^	1623 ± 148 ^b^	−13.2 ± 0.7 ^c^	−17.9 ± 1.4 ^c^
	3	2374 ± 117 ^b^	1287 ± 176 ^b^	−11.7 ± 0.5 ^c^	−18.4 ± 0.3 ^c^

Superscript letters indicate statistically significant differences (*p* ≤ 0.05) between the different samples of the double emulsion systems for each digestion phase. Analyses were conducted in triplicate (n = 3). * Content of *Opuntia ficus-indica* fruit pulp green extract in the emulsions is as follows: 1 refers to 1 g of OFC pulp green extract; 2 refers to 2 g of OFC pulp green extract; and 3 refers to 3 g of OFC pulp green extract. ^1^ TW: double emulsion systems based on Tween 20. ^2^ SC: double emulsion systems based on sodium caseinate.

**Table 7:** Digestive stability (µg of compound/emulsion) of the main bioactives in *O. ficus-indica* var. *Colorada* fruit pulps extracts encapsulated by TW and SC double emulsion systems during in vitro gastro-intestinal digestion.

Compound	Digestive Stability (µg of Compound/Emulsion)							
	Digestion Phase	OFC Pulp Extract	TW Double Emulsions ^2^*			SC Double Emulsions ^3^*		
			TW1	TW2	TW3	SC1	SC2	SC3
Indicaxanthin	Control ^1^	508 ± 10 ^a^	243 ± 34 ^a^	850 ± 40 ^b^	1366 ± 16 ^c^	257 ± 20 ^a^	670 ± 71 ^b^	1444 ± 70 ^c^
	Oral	389 ± 3 ^a^	187 ± 9 ^a^	802 ± 52 ^c^	1203 ± 30 ^c^	168 ± 19 ^a^	596 ± 34 ^c^	1135 ± 131 ^d^
	Gastric	306 ± 9 ^a^	176 ± 8 ^a^	696 ± 42 ^c^	1001 ± 16 ^d^	177 ± 49 ^a^	499 ± 14 ^b^	739 ± 30 ^c^
	Intestinal	323 ± 11 ^a^	155 ± 13 ^a^	700 ± 26 ^c^	996 ± 18 ^d^	162 ± 10 ^a^	370 ± 38 ^b^	694 ± 18 ^c^
Piscidic acid	Control ^1^	2641 ± 92 ^a^	1694 ± 75 ^a^	4753 ± 210 ^b^	11,449 ± 156 ^d^	1614 ± 176 ^a^	6248 ± 356 ^c^	11,345 ± 436 ^d^
	Oral	1112 ± 116 ^a^	930 ± 84 ^a^	3056 ± 290 ^b^	8443 ± 252 ^d^	914 ± 104 ^a^	4226 ± 222 ^c^	8876 ± 177 ^d^
	Gastric	247 ± 90 ^a^	891 ± 99 ^a^	2300 ± 296 ^b^	8434 ± 117 ^d^	701 ± 125 ^a^	4256 ± 326 ^c^	3493 ± 266 ^c^
	Intestinal	586 ± 79 ^a^	1174 ± 67 ^a^	3765 ± 198 ^b^	8853 ± 145 ^c^	707 ± 87 ^a^	4580 ± 226 ^b^	3345 ± 138 ^b^
4-hydroxibenzoic acid derivative (4-HAD)	Control ^1^	117 ± 6 ^a^	85.4 ± 2 ^a^	295 ± 9 ^b^	471 ± 16 ^c^	62.9 ± 2 ^a^	214 ± 57 ^b^	285 ± 30 ^b^
	Oral	78.1 ± 4 ^a^	62.8 ± 4 ^a^	246 ± 32 ^b^	316 ± 30 ^d^	44.8 ± 5 ^a^	186 ± 10 ^b^	300 ± 11 ^d^
	Gastric	67.9 ± 2.2 ^a^	58.7 ± 1.2 ^a^	188 ± 20 ^b^	334 ± 16 ^c^	51 ± 4 ^a^	158 ± 8 ^b^	174 ± 3 ^b^
	Intestinal	35.8 ± 4.1 ^a^	54.6 ± 3.3 ^a^	185 ± 14 ^b^	260 ± 18 ^c^	32.2 ± 2.1 ^a^	126 ± 4 ^b^	142 ± 8 ^b^
Isorhamnetin glucoxyl-rhamnosyl-pentoside 2 (IG2)	Control ^1^	31.9 ± 1.4 ^a^	28.9 ± 4.2 ^a^	41.4 ± 1.4 ^a^	50.8 ± 7.1 ^a^	17.6 ± 1.5 ^a^	40.3 ± 2.8 ^a^	60.4 ± 1.2 ^b^
	Oral	24.6 ± 2.6 ^a^	18.1 ± 1.2 ^a^	28 ± 1.1 ^a^	26.9 ± 0.8 ^a^	17.4 ± 0.5 ^a^	35.4 ± 3 ^a^	53.3 ± 6.1 ^b^
	Gastric	13.7 ± 0.2 ^a^	20.4 ± 0.8 ^a^	25.9 ± 1.3 ^a^	21.2 ± 1.7 ^a^	13 ± 0.7 ^a^	19.9 ± 1 ^a^	48 ± 4.9 ^b^
	Intestinal	8.3 ± 0.3 ^a^	16 ± 1.9 ^b^	26.5 ± 2 ^b^	32 ± 3.3 ^c^	8.1 ± 0.3 ^a^	18.7 ± 2.3 ^b^	37.5 ± 3 ^c^

Superscript letters indicate statistically significant differences (*p* ≤ 0.05) between the non-encapsulated OFC pulp green extract and the different samples of TW and SC double emulsion systems for the individual compounds analyzed in each digestion phase. Analyses were conducted in triplicate (n = 3). ^1^ Control refers to the samples before digestion. ^2^ TW: double emulsion system based on Tween 20. ^3^ SC: double emulsion system based on sodium caseinate. * 1 refers to 1 g of OFC pulp green extract; 2 refers to 2 g of OFC pulp green extract; and 3 refers to 3 g of OFC pulp green extract.

**Table 8:** Bioaccessibility (%) of the main bioactive compounds from *Opuntia ficus-indica* var. *Colorada* pulp green extracts encapsulated in TW and SC double emulsions after in vitro gastro-intestinal digestion.

Compound	Bioaccessibility (%)						
	OFC Pulp Extract	TW ^1^*			SC ^2^*		
		TW1	TW2	TW3	SC1	SC2	SC3
Indicaxanthin	40.9 ± 3.6 ^a^	63.8 ± 5.3 ^a^	82.3 ± 6 ^b^	72.9 ± 1.3 ^b^	63 ± 3.4 ^a^	55.2 ± 5.7 ^a^	48.1 ± 1.2 ^a^
Piscidic acid	43.6 ± 2.2 ^a^	80.7 ± 2.5 ^c^	82.8 ± 1.5 ^c^	79.1 ± 1.2 ^c^	64.6 ± 3.3 ^b^	76.9 ± 3.1 ^c^	35.2 ± 1.1 ^a^
4-hydroxibenzoic acid derivative (4-HAD)	30.6 ± 3.5 ^a^	63.9 ± 3.9 ^b^	62.8 ± 3.8 ^b^	55.3 ± 3.8 ^b^	51.2 ± 3.3 ^b^	58.9 ± 1.9 ^b^	49.7 ± 2.8 ^b^
Isorhamnetin glucoxyl-rhamnosyl-pentoside 2 (IG2)	25.9 ± 0.9 ^a^	55.2 ± 6.6 ^b^	64 ± 7 ^b^	63.1 ± 6.5 ^b^	45.5 ± 1.7 ^b^	46.3 ± 5.7 ^b^	62.1 ± 5 ^b^

Superscript letters indicate statistically significant differences (*p* ≤ 0.05) between the non-encapsulated OFC pulp green extract and the different samples of TW and SC double emulsion systems for the individual compounds analyzed in each digestion phase. Analyses were conducted in triplicate (n = 3). ^1^ TW: Double emulsion system based on Tween 20. ^2^ SC: Double emulsion system based on sodium caseinate. * 1 refers to 1 g of OFC pulp green extract; 2 refers to 2 g of OFC pulp green extract; and 3 refers to 3 g of OFC pulp green extract.

## Data Availability

The original contributions presented in the study are included in the article/Supplementary Material, further inquiries can be directed to the corresponding author.

## Supplementary Materials

The following supporting information can be downloaded at https://www.mdpi.com/article/10.3390/foods13071003/s1; Table S1: Composition of the digestive phases and enzymes used for the in vitro gastrointestinal digestion assay; Table S2: Antioxidant capacity of *Opuntia ficus-indica* var. *Colorada* pulp extracts and individual standards by the methods LOX-FL, ORAC and TEAC; Table S3: Particle size (nm) and zeta potential (mV) of TW and SC double emulsion systems with encapsulated extracts from *O. ficus-indica* var. *Colorada* pulps during 20 days conservation at 7 °C; Figure S1: HPLC C18 chromatograms of betalains and phenolic compounds from *Opuntia ficus-indica* var. *Colorada*, analysed at 480 nm (betaxanthins), 535 nm (betacyanins), 370 nm (flavonoids) and 280 nm (phenolic acids) wavelengths, where a) belongs to the non-encapsulated OFC pulp extract and b) to the encapsulated OFC pulp green extract in TW2 double emulsion system (based on Tween 20, containing 2 g of OFC pulp green extract); Figure S2. Inhibition of the LOX-1 dependent fluorescein bleaching by indicaxanthin, piscidic acid and isorhamnetin-glucoxyl-rhamnosyl-pentoside 2 (IG2); Figure S3. Visual inspection of the double emulsions systems based on Tween 20 (TW) and sodium caseinate (SC) with encapsulated *O. ficus-indica* var. *Colorada* pulp extracts during 20 days storage at 7 °C.
