# Supplementary material for: Encapsulation of Indicaxanthin-Rich Opuntia Green Extracts by Double Emulsions for Improved Stability and Bioaccessibility
Source: Foods. 2024 Mar 25;13(7):1003. doi: 10.3390/foods13071003 (PMC11012069; doi:10.3390/foods13071003)
Supplement: Supplementary file 1 [file foods-13-01003-s001.zip › foods-2931112-supplementary.pdf]

Table S1. Composition of the digestive phases and enzymes used for the *in vitro* gastrointestinal digestion assay.

Simulated digestive phases at 1:1 (w/w)				
**Reagents**	**Stock**	**Simulated oral phase**	**Simulated gastric phase**	**Simulated intestinal phase**
		**% (v/v)**		
KCl	0.5 M	2	5.6	2.16
KH_2PO_4	0.5 M	4	0.18	0.32
$NaHCO_3$	1 M	0.8	2.6	17
NaCl	2 M	0.2	2	3.2
$MgCl_2(H_2O)_6$	0.15 M	0.2	0.4	0.44
HCl	1 M	0.8	0.6	0.2
NaOH	1 M	0.2	-	0.32
MilliQ water	-	91.8	88.62	76.36
pH		6.8	1.3	8.2
**Addition of enzyme % (v/v)**				
Amylase	75 U/ml	5	-	-
Pepsine	2500 U/ml	-	4-8*	-
Lipase	25 U/ml	-	4	-
Pancreatin	100 U/ml	-	-	12
Bile salt	10 Mm	-	-	6

Table S2. Antioxidant capacity of *Opuntia ficus-indica* var. *Colorada* pulp extracts and individual standards by the methods LOX-FL, ORAC and TEAC.

**Sample**	**Antioxidant capacity***		
	LOX-FL (µmol Trolox eq/mg)	ORAC (µmol Trolox eq/mg)	TEAC (µmol Trolox eq/mg)
***Opuntia ficus-indica* var. *Colorada* pulp extract**	3.7 ± 0.2	51.6 ± 1.9	48.4 ± 2.1
**Indicaxanthin**	104 ± 2	17.3 ± 0.7	9 ± 2.1
**Piscidic acid**	0.29 ± 0.0	3.56 ± 0.2	6.43 ± 0.74
**Isorhamnetin glucosyl-rhamnosyl-pentoside 2 (IG2)**	1.9 ± 0.1	30.2 ± 1.2	140 ± 7

*Based on the study previously published by Gómez-Maqueo et al. 2021.

Table S3. Particle size (nm) and zeta potential (mV) of TW and SC double emulsion systems with encapsulated extracts from *O. ficus-indica* var. *Colorada* pulps during 20 days conservation at 7ºC.

Conservation	OFC pulp extract content*	Particle size (nm)		Zeta potential (mV)	
		TW[1]	SC	TW[1]	SC
Day 0	1	283 ± 10[a]	2694 ± 42[b]	-34.7 ± 2.4[a]	-43.2 ± 4.8[a]
	2	297 ± 21[a]	2286 ± 64[b]	-40.6 ± 1.3[a]	-38.2 ± 0.9[a]
	3	368 ± 8[a]	2622 ± 86[b]	-34.8 ± 2.5[a]	-39.5 ± 0.7[a]
Day 1	1	265 ± 7[a]	2821 ± 73[b]	-37.4 ± 2.1[a]	-46.2 ± 0.3[a]
	2	376 ± 12[a]	3190 ± 8[b]	-38.5 ± 1.1[a]	-38.7 ± 1.2[a]
	3	330 ± 2[a]	2989 ± 33[b]	-33.6 ± 5.5[a]	-40.4 ± 1.3[a]
Day 3	1	266 ± 4[a]	2911 ± 25[b]	-33.9 ± 0.5[a]	-41.7 ± 1.1[a]
	2	387 ± 4[a]	2977 ± 87[b]	-32.7 ± 0.4[a]	-37.6 ± 0.2[a]
	3	352 ± 9[a]	3008 ± 11[b]	-34.2 ± 3.9[a]	-35.5 ± 1.3[a]
Day 5	1	265 ± 3[a]	3267 ± 42[b]	-32.8 ± 2.1[a]	-38.5 ± 0.4[a]
	2	236 ± 4[a]	3373 ± 64[b]	-31.2 ± 0.5[a]	-37.4 ± 0.3[a]
	3	331 ± 5[a]	-	-34.1 ± 3.4[a]	-
Day 10	1	253 ± 1[a]	-	-24.8 ± 0.7[a]	-
	2	215 ± 4[a]	-	-29.0 ± 0.1[a]	-
	3	314 ± 3[a]	-	-33.4 ± 0.6[a]	-
Day 15	1	270 ± 4[a]	-	-26.0 ± 1[a]	-
	2	236 ± 4[a]	-	-21.8 ± 0.6[a]	-
	3	338 ± 2[a]	-	-31 ± 0.3[a]	-
Day 20	1	295 ± 7[a]	-	-25.9 ± 0.8[a]	-
	2	327 ± 5[a]	-	-22.2 ± 0.6[a]	-
	3	389 ± 7[a]	-	-29 ± 0.23[a]	-

Superscript letters indicate statistically significant differences ($p \leq 0.05$) between the different samples of the double emulsion systems on storage. Analysis were conducted in triplicate (n= 3).
*Content of *Opuntia ficus-indica* fruit pulp extract in the emulsions as follows: (1) 1 g of OFC pulp extract; (2) 2 g of OFC pulp extract; and (3) 3 g of OFC pulp extract

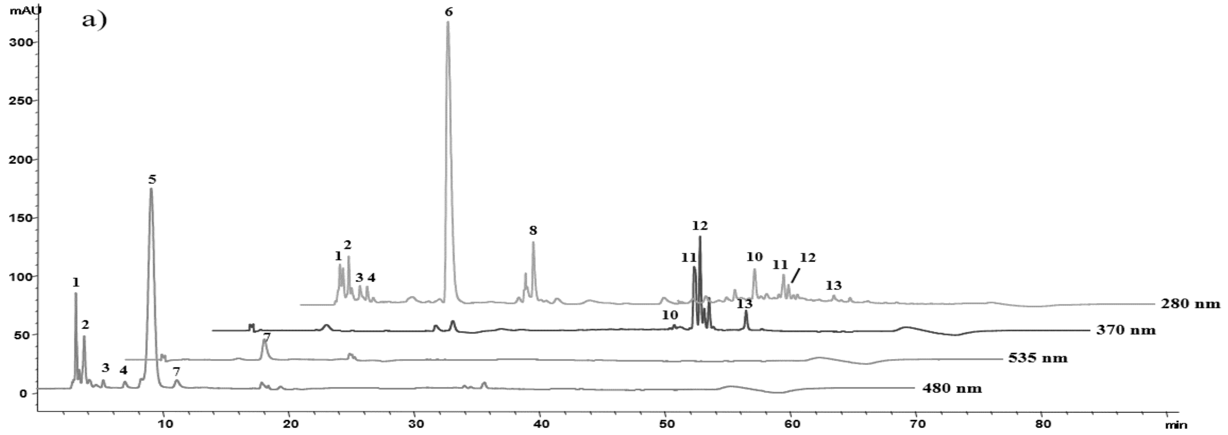

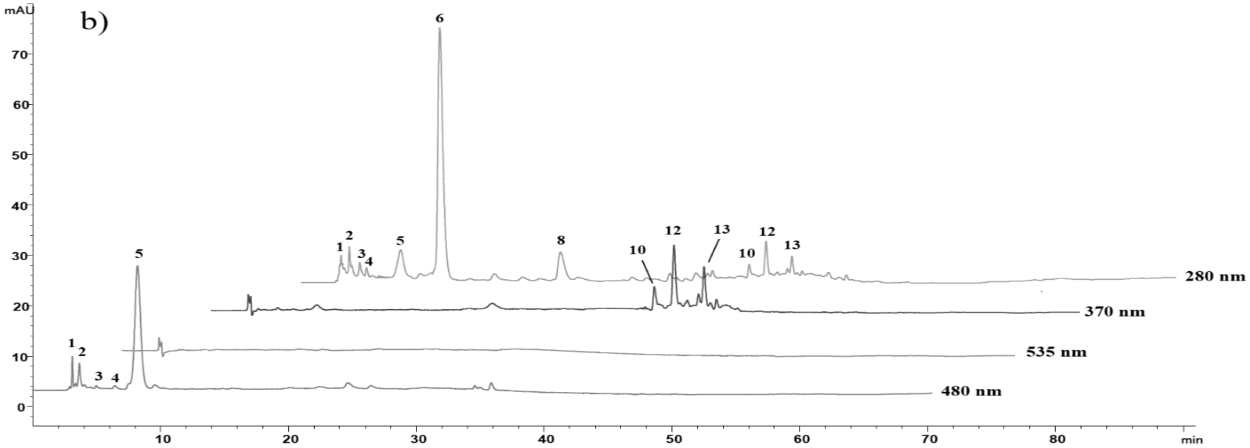

Figure S1. HPLC C18 chromatograms of betalains and phenolic compounds from *Opuntia ficus-indica* var. *Colorada*, analysed at 480 nm (betaxanthins), 535 nm (betacyanins), 370 nm (flavonoids) and 280 nm (phenolic acids) wavelengths, where a) belongs to the non-encapsulated OFC pulp extract and b) to the encapsulated OFC pulp green extract

in TW2 double emulsion system (based on Tween 20, containing 2 g of OFC pulp green extract).

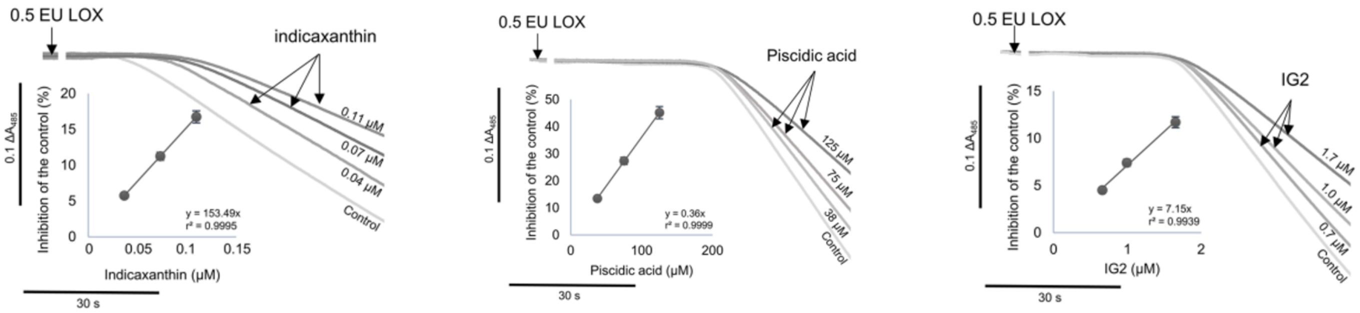

Figure S2. Inhibition of the LOX-FL by indicaxanthin, piscidic acid and isorhamnetin-glucoxyl-rhamnosyl-pentoside 2 (IG2).
*Based on the study previously published by Gómez-Maqueo et al. 2021.

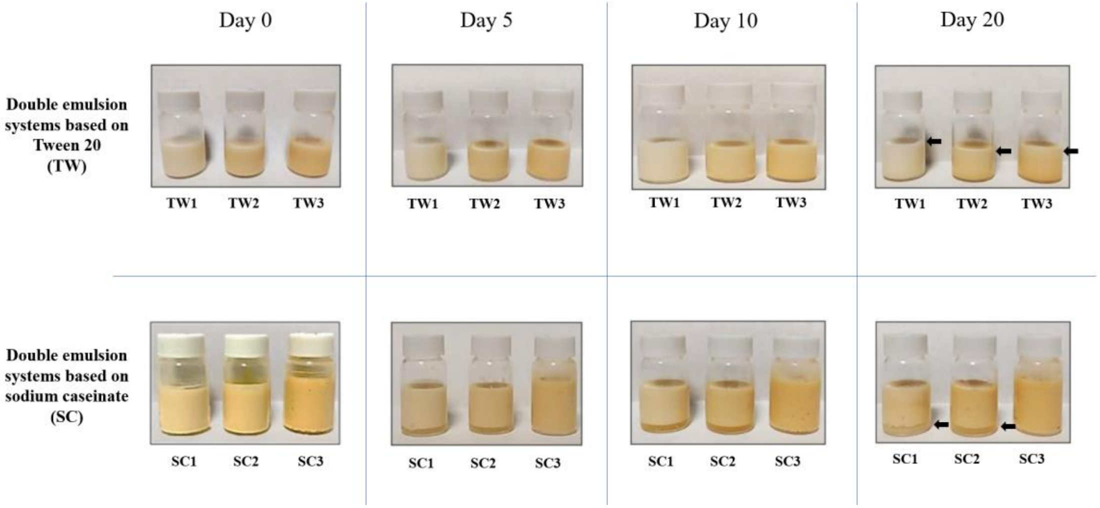

Figure S3. Visual inspection of the double emulsions systems based on Tween 20 (TW) and sodium caseinate (SC) with encapsulated *O. ficus-indica* var. *Colorada* pulp extracts during 20 days storage at 7ºC.
